# Multi-receptor skin with highly sensitive tele-perception somatosensory

**DOI:** 10.1126/sciadv.adp8681

**Published:** 2024-09-11

**Authors:** Yan Du, Penghui Shen, Houfang Liu, Yuyang Zhang, Luyao Jia, Xiong Pu, Feiyao Yang, Tianling Ren, Daping Chu, Zhonglin Wang, Di Wei

**Affiliations:** ^1^ Beijing Institute of Nanoenergy and Nanosystems, Chinese Academy of Sciences, Beijing 101400, China.; ^2^ School of Nanoscience and Engineering, University of Chinese Academy of Sciences, Beijing 100049, China.; ^3^ School of Integrated Circuits and Beijing National Research Center for Information Science and Technology, Tsinghua University, Beijing 10084, China.; ^4^ The University of Manchester, Manchester M13 9PL, UK.; ^5^ Centre for Photonic Devices and Sensors, University of Cambridge, 9 JJ Thomson Avenue, Cambridge CB3 0FA, UK.; ^6^ Guangzhou Institute of Blue Energy, Knowledge City, Huangpu District, Guangzhou 510555, China.; ^7^ Georgia Institute of Technology, Atlanta, GA 30332-0245, USA.

## Abstract

The limitations and complexity of traditional noncontact sensors in terms of sensitivity and threshold settings pose great challenges to extend the traditional five human senses. Here, we propose tele-perception to enhance human perception and cognition beyond these conventional noncontact sensors. Our bionic multi-receptor skin employs structured doping of inorganic nanoparticles to enhance the local electric field, coupled with advanced deep learning algorithms, achieving a Δ*V*/Δ*d* sensitivity of 14.2, surpassing benchmarks. This enables precise remote control of surveillance systems and robotic manipulators. Our long short-term memory–based adaptive pulse identification achieves 99.56% accuracy in material identification with accelerated processing speeds. In addition, we demonstrate the feasibility of using a two-dimensional (2D) sensor matrix to integrate real object scan data into a convolutional neural network to accurately discriminate the shape and material of 3D objects. This promises transformative advances in human-computer interaction and neuromorphic computing.

## Teaser

Multi-receptor skin uses inorganic nanoparticles and deep learning to effectively enhance tele-perception and tactile perception.

## INTRODUCTION

**Fig. 1.**
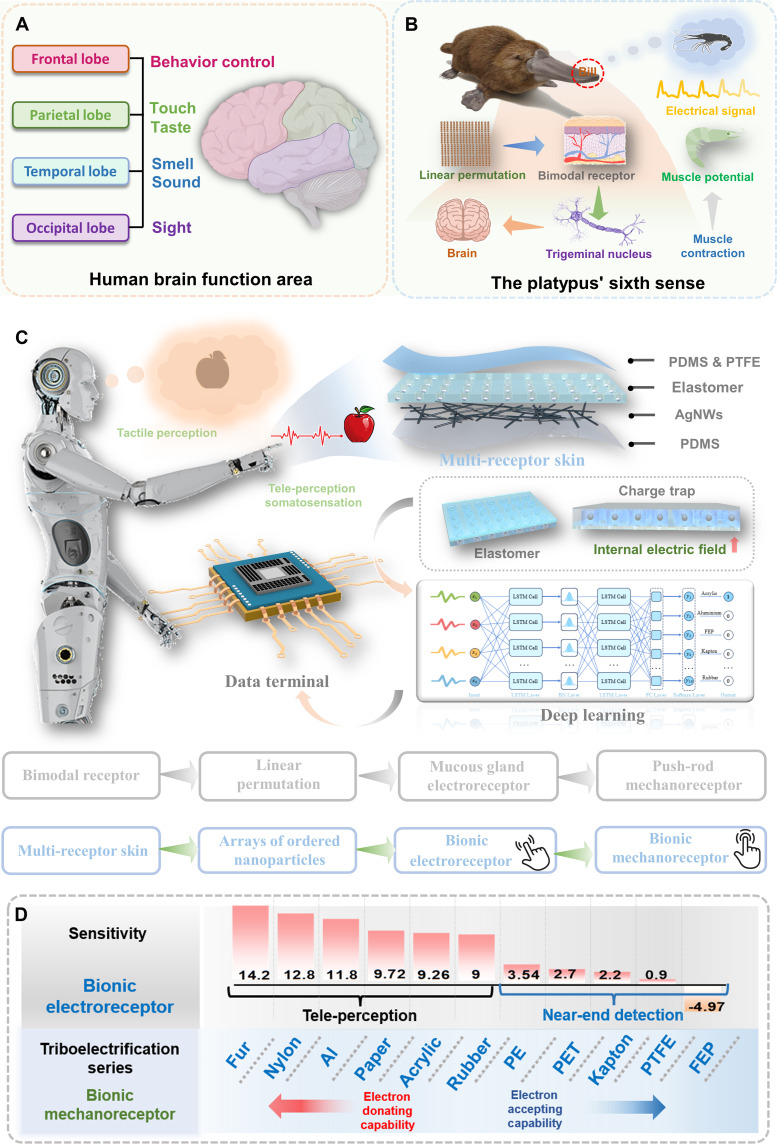
Multi-receptor skin perception system. (**A**) Human brain function area. (**B**) Schematic demonstration of the dual receptor system that is distributed on the platypus’s bill for environmental perception. (**C**) Schematic diagram of intelligent perception system based on the multi-receptor skin and deep learning. (**D**) The applicability of the multi-receptor (bionic electroreceptor and bionic mechanoreceptor) skin for different materials.

Humans perceive the world through multi-sensory (touch, sight, sound, taste, and smell) integration. Recent advances in humanoid robots (*1*–*3*) and human-machine interface (HMI) (*4*, *5*) underscore an urgent imperative to amplify the human sensory apparatus (*6*–*8*) (Fig. 1A) and potentially extend beyond the conventional five senses to encompass the elusive sixth sense. Tele-perception represents a paradigm shift in human cognition, with considerable potential for enhancing situational awareness, decision-making, and environmental interaction. By surpassing the limitations of traditional senses, tele-perception provides a pathway to unlock innovative dimensions of human perception and cognition. Current tactile sensors (*9*–*11*), essential for intelligent perception and control, such as pressure-sensitive arrays (*12*–*14*), pliant optical strain sensors (*15*), magnetic micro-electro-mechanical sensors (*16*–*18*), capacitive arrays (*19*–*21*), and piezoelectric sensors (*22*–*24*), rely on physical contact and encounter limitations in perceiving objects without direct interaction, constraining human-robot interaction framework (*25*, *26*). Efforts have focused on enhancing precontact capabilities through innovations in surface structuring (*27*, *28*), the employment of composite materials (*29*–*31*), ion injection (*32*), and the creation of electrets imbued with surface charges (*33*). However, the deficient charge capture capacity inherent in the dielectric layer further compromises sensitivity (*34*, *35*). While precontact somatosensation, as defined through simulation modeling, can achieve three-dimensional (3D) shape identification, the limited sensitivity of electroreceptors inhibits simultaneous identification of both material and shape (*36*), which persists as a formidable challenge. Therefore, integrating tactile perception and tele-perception functionalities into advanced sensors is pivotal (*37*, *38*), leveraging progress in materials science, nanotechnology, and deep learning algorithms (*39*–*41*). Key strategies involve enhancing charge capture capacity to advancing tele-perception somatosensation (*42*, *43*).

Biological systems present an abundance of exemplary templates, particularly within domains associated with perception and tactile discernment. Notably, the platypus (*44*) serves as an exemplar, possessing specialized mechanoreceptors and electroreceptors orderly arranged on its bills (*45*, *46*), creating a series of parallel stripes. This elaborate arrangement of thousands of dual receptors adeptly responds to both mechanical and electric stimuli, enabling a comprehensive reaction to natural stimuli. For instance, when a shrimp’s tail initiates muscle contractions, concurrently generating electric signals and mechanical disturbances, the platypus’s distributed electroreceptors within the soft dermal layer discern faint bioelectric signals correlated with prey muscle contractions, while mechanoreceptors promptly respond to stimuli associated with physical contact. The dynamic sensitivity to efficacious stimuli is intricately regulated by the voltage drop across the skin. The pulse activation process of the receptor involves the polarization of the axon membrane, and these consequential changes in membrane potential are transmitted as pulse signals to the brain via the trigeminal nerve. This intricate mechanism facilitates the platypus in various tasks, including detection, communication, hunting, and navigation (Fig. 1B).

Inspired from the dual sensory system and structured arrangement of receptors observed in the platypus for detection and navigation, we propose the concept of tele-perception to expand human perception and cognition, surpassing the constraints of noncontact sensors and traditional sensory modalities. Our designed bio-inspired multi-receptor skin achieves superior tele-perception and tactile perception through structured incorporation of inorganic nonmetal nanoparticles and integration with advanced deep learning algorithms. On the basis of experiments and COMSOL (Comsol Multiphysics) simulation, the charge trap mechanism is proposed, and it is initiated by induced polarization facilitated by the structured doping of inorganic nonmetal nanoparticles to amplify local electric fields. This mechanism enhances tele-perception somatosensation and establishes a record-high sensitivity benchmark (Δ*V*/Δ*d* = 14.2), surpassing previously reported standards by an order of magnitude, which enables precise remote control over surveillance system and robotic manipulators. Another breakthrough involves the development of an enhanced artificial intelligence algorithm designed for harsh environments, aimed at improving its robustness, adaptability, and accuracy. This algorithm integrates adaptive pulse identification technology with the inherent memory efficiency of the long short-term memory (LSTM) algorithm. When conjoined with the multi-receptor skin, tactile perception achieves 99.56% accuracy on materials identification, accompanied by accelerated processing speeds. It achieves an impressive CPU inference time of just 0.04392 s—25 times faster than conventional CNN models. Furthermore, we have demonstrated the feasibility of integrating data obtained from real object scans using a 2D sensor matrix into a convolutional neural network (CNN), thereby facilitating the development of a multi-receptor skin with tele-perception somatosensation for real-world applications. The flexible AI-driven e-skin composed of nano-materials ushers in capabilities in exteroceptive tele-perception and proprioceptive tactile perception, promising transformative advancements in various fields such as human-machine interaction and neuromorphic computing.

## RESULTS

### Design of the multi-receptor skin perception system

Inspired from the dual sensory system and the systematically structured arrangement of receptors observed in the platypus for detection and navigation (Fig. 1B), we have devised a multi-receptor (bionic electroreceptor and bionic mechanoreceptor) skin based on structured doping of inorganic nonmetal nanoparticles, tailored for tele-perception and tactile perception, respectively. The design inspiration on the process diagram for the multi-receptor skin is illustrated in Fig. 1C. The creation of the multi-receptor skin entailed the fusion of micro-nanofabrication techniques with structured doping of inorganic nonmetal nanoparticles. Initially, a meticulously designed silicon wafer template with regular microstructures (fig. S1) was crafted using ultraviolet photolithography. Subsequently, a precursor of polydimethylsiloxane (PDMS) doped with polytetrafluoroethylene (PTFE) emulsion (fig. S2) was cast onto the wafer template to produce a thin film featuring a micro-hole array. Inorganic nonmetal nanoparticles (SrTiO_3_) were then embedded in an elastomer possessing a microporous array. Using bonding techniques, a PTFE & PDMS film was overlaid onto the microholes, resulting in an elastomer characterized by a structured arrangement of inorganic nonmetal nanoparticles. Detailed experimental procedures and characteristics were delineated in Materials and Methods and the Supplementary Materials (fig. S3). Scanning electron microscopy (SEM) images of the corresponding materials synthesized by micro and nano processing are shown in figs. S4 to S6. During the nanoparticle introduction stage, high-speed spin-coating played a key role. By rotating at high speeds, nanoparticles in the precursor solution were evenly distributed at the bottom of the micropores in the elastomer. This technique not only enhanced the dispersibility and uniformity of the particles but also effectively controlled the coating thickness and surface smoothness. The application of high-speed spin-coating (*47*, *48*), particularly in the preparation of microstructured materials, substantially reduced issues of inhomogeneity caused by particle agglomeration, thereby improving overall structural consistency and performance stability. The structured doping of inorganic nonmetal nanoparticles, which induced polarization under external electric field precharging, promotes the generation of charge traps within the elastomer, resulting in the establishment of uniform and ordered electric fields, thereby enhancing the performance of the multi-receptor skin. The structural configuration of the multi-receptor skin adopted a single-electrode design, as depicted in Fig. 1C. This architecture comprised a PTFE & PDMS thin film, a layer of elastomer with an structured doping of inorganic nonmetal nanoparticles, a layer of silver nanowires (AgNWs) serving as the electrode, and a PDMS-encapsulated substrate. The AgNW electrode acts as a collector for the multi-receptor skin, and its main role is to derive the output voltage of the device. A schematic diagram of an intelligent perception system based on the multi-receptor skin and the deep learning algorithm is elucidated in Fig. 1C. The multi-receptor skin seamlessly integrated hybrid mechanisms derived from a multi-sensory system, encompassing tele-perception somatosensation and tactile perception for material identification. The signals generated by the multi-receptor skin underwent meticulous data collection, preprocessing, and neural network learning. The trained neural network was then deployed in the testing system.

Figure 1D displays the applicability of the multi-receptor skin for different materials. Like the remote perception capability naturally evolved in the platypus, the skin exhibited the highest activity and threshold voltage when a positively charged object approached the center of its bimodal receptors. This unique monotropic remote (*49*) perception mechanism provides a crucial advantage for the platypus in surviving and reproducing in complex aquatic environments. Furthermore, unipolar perception capability has superior polarization characteristics and electric field perception ability, allowing for more precise detection of changes in the electrical signals of charged objects. This enhances the sensitivity and accuracy of the multi-receptor skin. In an emulation of the platypus’s monotropic characteristics, the triboelectric properties of the engineered multi-receptor skin manifest a pronounced electronegativity in the triboelectric series, positioning itself roughly between PTFE and fluorinated ethylene propylene (FEP). The bionic electroreceptor demonstrated proficiency in tele-perception somatosensation, effectively locating targets by generating electric signals upon the appearance of the anticipated target. The variations in electronegativity and tele-perception triboelectrification capabilities with different materials resulted in diverse sensitivities exhibited by the multi-receptor skin. The tele-perception achieved an unprecedented sensitivity record (Δ*V*/Δ*d* = 14.2), surpassing the benchmarks hitherto reported by an order of magnitude. Furthermore, the bionic mechanoreceptor served for tactile perception in material identification and the design of contact human-machine tactile interactive interfaces. Signals generated by touching materials were collected and subjected to deep learning in the testing system for material classification. This integration enabled the multi-receptor skin to have both exteroceptive tele-perception and proprioceptive tactile perception capabilities. This paradigm shift offers a pioneering research avenue for surpassing human tactile capabilities and efficiently detecting tele-perception events in soft robotic sensors or interfaces.

### Mechanism and the simulation on the multi-receptor skin

**Fig. 2.**
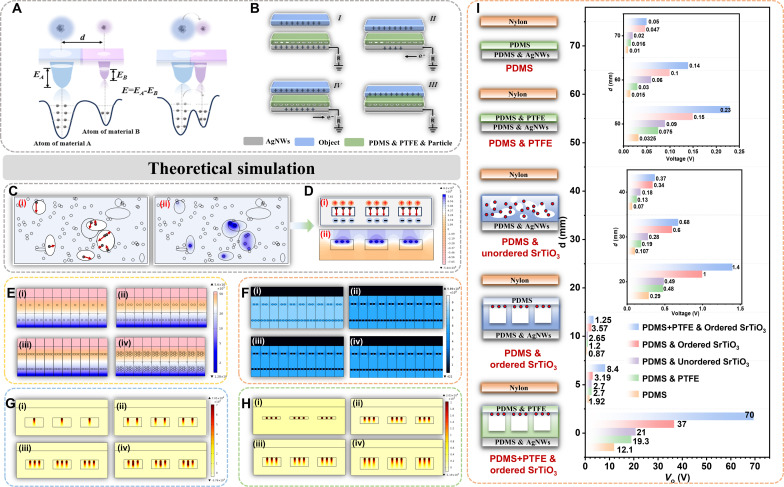
Mechanism and the simulation on the multi-receptor skin. (**A**) The basic principles of tactile perception explained by the overlapping electron cloud model. (**B**) The bionic electroreceptor working mechanism for tele-perception. (**C**) (i) Schematic diagram of disordered doping and (**D**) (i) structured doping between inorganic nonmetal nanoparticles and micropores. (C) (ii) Potential distribution across disordered doping and (D) (ii) structured doping between inorganic nonmetal nanoparticles and micropores under open-circuit conditions evaluated by COMSOL. (**E**) Potential distribution across PDMS doped with PTFE emulsions of different mass fractions [(i) 3%, (ii) 5%, (iii) 10%, and (iv) 20%] and (**F**) different particles [(i) SiO_2_, (ii) TiO_2_, (iii) BaTiO_3_, and (iv) SrTiO_3_] under open-circuit conditions evaluated by COMSOL. (**G**) Electric field distribution across PDMS doped with SrTiO_3_ particles with different mass fractions [(i) 4%, (ii) 8%, (iii) 12%, and (iv) 16%] and (**H**) PDMS with different micropore depths [(i) 10 μm, (ii) 25 μm, (iii) 40 μm, and (iv) 50 μm] under open-circuit conditions evaluated by COMSOL. (**I**) Experimental verification of the effectiveness of structured doping of inorganic nonmetal nanoparticles and micropores.

The phenomenon of tactile perception based on contact electrification can be elucidated through the model of overlapping electron clouds, providing insights into the underlying principles governing the reception of electric signals by the multi-receptor skin. As depicted in Fig. 2A, before the contact of two materials at the atomic scale, their respective electron clouds exist independently, devoid of any overlapping regions. The potential well tightly confines electrons to specific orbits, impeding electron transfer. Upon close contact, the electron clouds of the two atoms overlap, forming a bond. The application of an external force results in the shortening of the bond length, inducing a transition from the initial single-well potential to an asymmetric double-well potential. The strong overlap of electron clouds reduces the energy barrier between atoms, facilitating the transfer of electrons from one atom to another, generating triboelectric electricity. On the other hand, the bionic electroreceptor working mechanism for tele-perception is illustrated in Fig. 2B, involving the combination of triboelectric electricity and electrostatic induction. This mechanism can be delineated into four main steps. During relative motion with a charged object, electrons are transferred, generating charge differences on the object’s surface. Further details of the working mechanism are elucidated in fig. S7. Following the precharging of the multi-receptor skin, dielectric polarization occurred based on the structured doping of inorganic nonmetal nanoparticles within the elastomer. As the positively charged nylon film approached the elastomer, it accumulated a relative positive charge on its surface. Simultaneously, the reduction in surface polarization resulted in the return of positive charges on the AgNW electrodes to the reference electrode until the nylon and the elastomer once again approached in proximity.

However, the presence of dielectric hysteresis ensures that the polarization inside the elastomer does not fully diminish. The residual embedded dielectric polarization serves as a negative charge trap, thereby augmenting the elastomer’s capacity to capture charges when in proximity to a charged object. Consequently, the multi-receptor skin’s efficacy in detecting electric signals during operation is substantially enhanced. Figure 2C(i) depicts a schematic diagram illustrating disordered doping between inorganic nonmetal nanoparticles and micropores. The supplementary files (texts S1 and S2) showcased the random generation of pore sizes and particle numbers using Java. The micropores exhibited varying sizes and shapes, and the arrangement of inorganic nonmetal nanoparticles within these micropores was random. Such chaotic arrangement has the potential to disrupt the direction of the internal triboelectric field and may even lead to the cancellation of the internal triboelectric field. In contrast, Fig. 2D(i) demonstrates the advantage of embedding inorganic nonmetal nanoparticles in the micropores in a uniform direction, forming a uniformly ordered electric field inside the elastomer. To theoretically predict the electric field distribution of disordered doping and structured doping, finite element analysis was conducted using COMSOL, as depicted in Fig. 2 [C(ii) and D(ii)]. The electric field of disordered doping was notably lower than that of structured doping due to mutual cancellation. To theoretically predict the parameters for optimal performance of structured doping, finite element analysis was again performed using COMSOL (Fig. 2, E to H). The results indicated that optimal performance was observed for the elastomer with a micropore depth of 40 μm, doped with a mass fraction of 10% PTFE emulsion and 12% SrTiO_3_ particles. Simultaneously, experimental validation is illustrated in the supplementary files (figs. S8 to S11), and the results are consistent with theoretical simulations. Furthermore, we chose SrTiO_3_ nanoparticles to investigate the charge trapping characteristics as particle size varies. The results indicate that the output voltage signal initially increases and subsequently decreases with increasing particle size, with optimal performance observed at a particle size of 50 nm. This phenomenon is attributed to larger nanoparticles potentially causing stress concentration within the microporous structure, which can lead to structural damage. Conversely, when nanoparticle size is too small, the specific surface area increases considerably. This may enhance surface energy, making the particles more active, but it can also result in excessive surface defects and instability, thereby affecting the material’s long-term stability and performance (fig. S12). Upon embedding nanoparticles into pores, interactions with pore surfaces prompt charge redistribution, potentially resulting in the entrapment of electrons or holes proximal to nanoparticle surfaces or interfaces, thus forming charge traps. The presence of such traps may augment local electric fields. Generally, particles with high dielectric constants manifest intensified electric fields, consequently optimizing signal output by virtue of their heightened electron capture efficacy. Furthermore, certain inorganic nonmetallic materials have intrinsic non-centrosymmetric crystal structures, which induce polarization when subjected to an external electric field. The charge traps formed by this unique property combined with the controlled micro-pore array structure effectively prevent charge escape and enhance the electric field, thereby endowing them with distinct advantages in exteroceptive tele-perception and proprioceptive tactile perception capabilities.

According to the triboelectric series of materials, PTFE acquires electrons more readily than PDMS. Consequently, PTFE particles would exhibit a higher electron density on the surface. With an increase in the PTFE particle content, a greater number of electrons were captured in both PDMS and PTFE samples. However, as the PTFE content increased from 10 to 20%, the PTFE particles within the PDMS matrix became substantially larger due to the preparation of the PDMS & PTFE composite film. Mathematically, it could be deduced that when the PTFE particle size became excessively large, the interface between PDMS and PTFE diminished in the surface region of the PDMS matrix, leading to a decrease in the induction of surface charge. In addition, SrTiO_3_ exhibited a stronger potential distribution due to its high dielectric constant. However, when the doping concentration of SrTiO_3_ was too high, a conductive path formed inside the elastomer, resulting in surface charge leakage. Conversely, if the pores were too small, internal triboelectrification decreased. When the pores were too deep, more defects were likely to form inside the elastomer. To validate the effectiveness of the structured doping of inorganic nonmetal nanoparticles in micropores (optimal parameters identified through simulation and experimentation), nylon was selected as the charged object for approaching. The elastomers considered for this evaluation included PDMS film, PDMS & PTFE composite film, disorderedly doped particle film with PDMS, orderly doped nanoparticle film with PDMS, and orderly doped nanoparticle film with PDMS & PTFE. The results demonstrate that the structured doping of inorganic nonmetal nanoparticles enhances the signal capture capability, and the output voltage exhibits optimal performance (Fig. 2I).

### Tele-perception somatosensation of the bionic electroreceptor

**Fig. 3.**
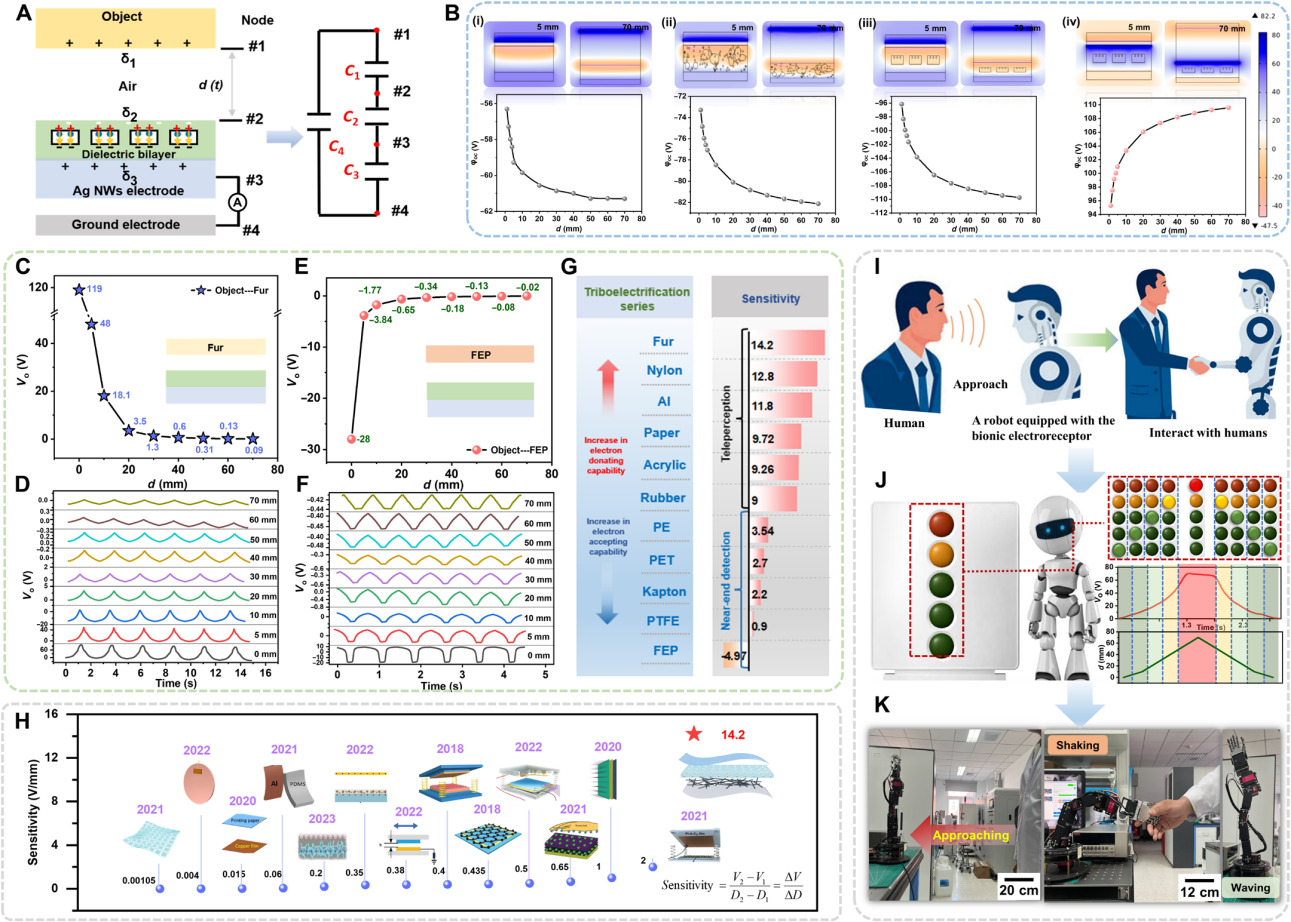
Tele-perception somatosensation of the bionic electroreceptor. (**A**) A simplified physical model of the bionic electroreceptor. (**B**) The behavior of the bionic electroreceptor was simulated through the finite element method. Fur (**C** and **D**) and FEP (**E** and **F**) films were selected as the output voltage of the perception target approaching bionic electroreceptor, respectively. (**G**) The applicability of the bionic electroreceptor for different materials. (**H**) A summary of the sensitivity electroreceptor and the representative work and the corresponding realization method of this work. (**I**) The application scenarios of the bionic electroreceptor in intelligent robotic systems are envisaged. (**J**) A virtual distance alarm robot based on the bionic electroreceptor. (**K**) Demonstration on how to operate a robotic arm to wave and shake hands when an adult approaches.

The electroreception process of the multi-receptor skin is elucidated using the simplified physical model depicted in Fig. 3A. This model encompasses a charged body, the upper surface of the elastomer, and two electrodes represented as nodes 1 to 4. An equivalent circuit with four capacitors (C_1_ to C_4_) was established, with C_1_ to C_3_ in series and C_4_ in parallel. Among these, C_3_ represents the parasitic capacitance of the triboelectric nanogenerator, where the bottom electrode of the bionic electroreceptor forms an air capacitance with the ambient air. Parasitic capacitance in the air often affects the frequency response and noise characteristics of the signal because it is not intentionally designed into the system but still impacts the measured signal. This can result in additional signal attenuation or introduce various types of noise, necessitating consideration during both design and analysis phases. Meanwhile, C_1_ and C_2_ may influence the output voltage during operation. Notably, only C_2_ was controllable and could optimize charge retention capacity. A detailed theoretical explanation of the behavior of the bionic electroreceptor was provided through finite element method simulations (Fig. 3B). The theoretical performance of the bionic electroreceptor was characterized by the curve of the potential content (φ_OC_) on the surface of the elastomer. Simulated voltage-displacement curves were plotted when objects with positive charges (nylon) approached the elastomer, including PDMS film [Fig. 3B(i)], disordered doped thin films of and PDMS [Fig. 3B(ii)], and structured doped film of particles and PDMS [Fig. 3B(iii)]. The simulation results demonstrated a stronger capability of capturing signals in the case of the structured doping of particles [Fig. 3B(iii)]. As the object (FEP) approached the elastomer, the FEP surface induced negative charges, explaining the opposite dynamic trend compared to the object (nylon) with positive charges [Fig. 3B(iv) and fig. S13]. Both the positively charged nylon and negatively charged FEP were chosen as charged objects. The tele-perception somatosensation capability of the bionic electroreceptor was then tested over a gap distance of 0 to 70 mm, demonstrating that the voltage (*V*_o_) closely tracked the target’s position (Fig. 3, C to F), consistent with simulation results. The voltage curves of the bionic electroreceptor after high-voltage polarization treatment are shown in fig. S14A. These curves clearly demonstrated the bionic electroreceptor’s stable signal capture trend. In contrast, if the bionic electroreceptor only experienced brief contact without high-voltage polarization, the signal exhibited an attenuation trend when the distance increased to 5 mm (fig. S14B). This was because the charge traps within the bionic electroreceptor were shallow and insufficient in number, leading to easy charge escape, which subsequently affected signal stability. This indicated that high-voltage polarization treatment played a crucial role in ensuring the sensor’s long-term stable operation and tele-perception capability. These experimental data validated the sensor’s ability to capture material characteristics at long distances, emphasizing its effectiveness and reliability in tele-perception. Given that most objects in our daily life carry charges, the electroreceptor is theoretically capable of detecting the vast majority of targets, an expectation verified by results in Fig. 3G. We experimented with 10 kinds of materials in our laboratory without further treatment, covering fur, FEP, nylon, PTFE, rubber, paper, acrylic, Al, polyethylene terephthalate (PET), Kapton, and polyethylene (PE), and all targets (fig. S15) were successfully sensed.

In addition, we selected two materials with different dielectric constants, A and B. Material A and material B were tightly fitted vertically (left-right) or tightly fitted vertically (top-down), respectively, to form a mixed material sample (fig. S16A). Electrical signals for material A and material B were measured separately using the bionic electroreceptor, and the data were recorded. The mixed material samples’ electrical signals were then measured and recorded using the bionic electroreceptor. In our experiments, we selected materials with small differences in electronegativity (nylon/Al), medium differences in electronegativity (nylon/rubber), and large differences in electronegativity (nylon/FEP) for validation (fig. S16B). The experimental results showed that the output signal of the mixed material was usually intermediate between the electrical signals of the two individual materials, regardless of which side of the mixed material A or B the bionic electroreceptor was vertically close to (fig. S16C). Similarly, the output voltage signals of the bionic electroreceptor in plane recognition of the mixed material are shown in fig. S16D and exhibit the same phenomenon. This suggests that the dielectric constant of the mixed material is jointly affected by the dielectric constants of the two materials. This phenomenon can be explained by dielectric theory. When two different materials are laminated, the dielectric constant of the mixed material is between the dielectric constants of material A and material B and varies according to the ratio and distribution of the two materials. The change in the dielectric constant of the mixed material leads to a change in its capacitive properties, which affects its electrical signal. With these measurements, we verified the effectiveness of the bionic electroreceptor in recognizing mixed materials. A summary of the sensitivity electroreceptor and the representative work and the corresponding realization method of this work is illustrated in Fig. 3H (*50*–*60*). The tele-perception achieved an unprecedented sensitivity record (Δ*V*/Δ*d* = 14.2). The sensitivity of the bionic electroreceptors and their representative work and the corresponding device parameters are shown in table S1. The tele-perception somatosensory capacity of the bionic electroreceptor was subsequently assessed as it approached fur over gap distances of 100 and 150 mm, yielding signals of 0.038 and 0.014 V, respectively (fig. S17). This showcases the sensitivity and operational efficacy of the bionic electroreceptor, setting a benchmark in comparison to previous precontact sensors. Moreover, under stretching conditions, the bionic electroreceptor exhibited minimal decrease in output voltage, meeting the requisites for flexible and stretchable devices (fig. S18). The performance of the bionic electroreceptor remained consistent during a 70-day storage period at room temperature, affirming its charge stability (fig. S19). Simultaneously, we conducted a comparative analysis of the bionic electroreceptor’s output under diverse environmental conditions {light and darkness, various temperatures, and various humidities [relative humidity (RH)]}. The results exhibited independence from ambient brightness within a specific humiture range (figs. S20 to S22). Furthermore, when placed in an environment with a temperature of 85°C and an RH of 85% for tele-perception perception within a 5-mm interval, the signal retention of the bionic electroreceptor remained at 93.5% even after 350,000 cycles, surpassing the performance of the PDMS film and the PDMS film with randomly dispersed inorganic nonmetal nanoparticles (fig. S23). This superiority is attributed to the high charge storage capability within the elastomer.

In Fig. 3I, a conceptual illustration delineated a pioneering tele-perception HMI scenario. A robot equipped with the bionic electroreceptor can detect human motion as a proximity sensor. Leveraging the sensitive and stable signal response, we developed a virtual distance alert robot interface using a customized LabVIEW program (Fig. 3J). The configuration and hardware configuration of the corresponding LabVIEW-based system are demonstrated in fig. S24 and text S3. The robot, equipped with a series of indicators, dynamically switched based on calibrated threshold voltages. When a target (nylon) approached, the real-time monitoring of the bionic electroreceptor’s output voltage would initiate, triggering the illumination of the corresponding indicator when reaching the predetermined threshold. The system was designed to transition from green to yellow when the distance is less than 10 mm, and to red when reduced to 5 mm, providing an intuitive warning. This process is highly responsive (movie S1). Notably, the threshold voltage can be adjusted to accommodate different materials, despite variations in the surface charge carried by approaching targets (fig. S25). The tele-perception interaction makes the electroreceptor appealing in the realms of robotics and HMIs. Subsequently, tele-perception in human-machine interaction was demonstrated using the bionic electroreceptor (Fig. 3K). Specifically, a signal generated by a nearby person was detected by the bionic electroreceptor, transmitted to the data acquisition board, and processed by a dedicated LabVIEW software program. The program analyzed the acquired signal and dispatched appropriate commands to the robotic arm (figs. S26 and S27). The voltage signal increased continuously as a person approached, and once it reached a precalibrated threshold, commands for waving and shaking hands are transmitted to the robot. In our experiments, the electronegativity of the elastomer in the bionic electroreceptor lay between PTFE and FEP. Three materials were selected with varying electronegativity differences relative to the elastomer: notably different (fur), moderately different (rubber), and slightly different (FEP). Experimental results showed distinct response characteristics of the bionic electroreceptor when detecting these three materials (fig. S28), with detailed parameters listed in table S2. In addition, the tele-perception interaction between a human wearing the material and a robotic arm is illustrated in fig. S29 and movie S2. Our tele-perception human-computer interaction system used the bionic electroreceptor to detect human electrical signals, with voltage thresholds set via the LabVIEW interface. When the detected signal reached or exceeded the threshold, the system triggered the robotic arm to interact with the individual. The adaptability of the threshold voltage through control enhances the appeal of tele-perception interaction in the fields of robotics and HMIs.

### Identification of materials by the bionic mechanoreceptor array

**Fig. 4.**
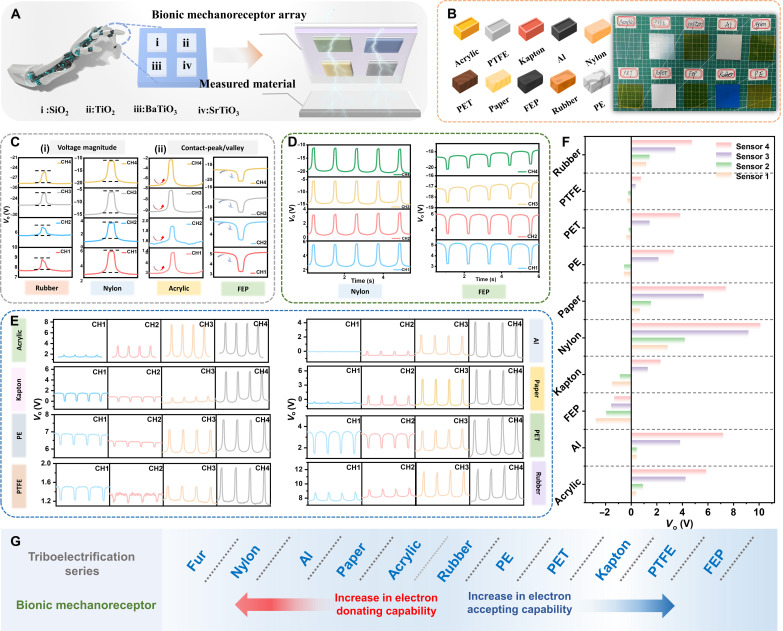
Identification of material types by the bionic mechanoreceptor array. (**A**) Schematic diagram of the bionic mechanoreceptor array. (**B**) Materials and photographs tested in the experiment. (**C**) Feedback characteristics of electric signals to different contact materials: (i) voltage amplitude and (ii) contact peak/valley. (**D**) Output voltage of bionic mechanoreceptor array in contact with positive and negative materials (nylon, FEP). (**E**) Bionic mechanoreceptor array detected the voltage output of acrylic, Kapton, PE, PTFE, Al, paper, PET, and rubber, respectively. (**F**) Normalized voltage characteristics of biomimetic mechanoreceptor arrays exposed to different materials. (**G**) Typical materials located in different positions in the triboelectric series.

On the basis of the elucidated operational principles of the bionic electroreceptor, a systematically designed 2 × 2 bionic mechanoreceptor array is presented in Fig. 4A. Each sensor within the array was infused with distinct polymer powders, designated as sensor 1, sensor 2, sensor 3, and sensor 4, respectively. Combining different sensors according to the triboelectric series allowed for a more comprehensive characterization of the subjected entity, thereby enhancing the precision of identification. The correlation between sensor spacing and output voltage is illustrated in fig. S30, demonstrating heightened discriminability of voltage signals at a spacing of 1 mm. The contact-separation process between the biomimetic mechanical sensor and the unknown material was equivalent to the complete charge transfer process under the single-electrode mode. Upon contact with the unidentified material, surface charges underwent transference due to disparate electron affinity capabilities. A basic testing simulator was designed, comprising a computer-structured linear motor simulating the effect of finger touch, a multi-channel data acquisition program for recording and storing data, and various commonly used materials with identical specifications to ensure universality, as illustrated in Fig. 4B. The first characteristic pertained to the voltage amplitude, with each material yielding distinct voltage values based on its electron affinity. Figure 4C(i) illustrates variations in the four voltage amplitudes of the biomimetic mechanical sensor array corresponding to the same material. The second characteristic involved the state of the contact signal curve, as depicted in Fig. 4C(ii). The inherent electron capture capability of the material also affected the polarity of the voltage signal. When in contact with the biomimetic mechanical sensor, acrylic manifested a positive voltage signal (contact peak), whereas FEP exhibited a negative voltage signal (contact valley). Furthermore, the sequencing of the four voltage amplitudes for each sensor in the biomimetic mechanical sensor array could serve as a distinctive characteristic for material identification. For instance, in the case of acrylic, the peak values followed the order sensor 4 > sensor 3 > sensor 2 > sensor 1 [Fig. 4C(ii)]. The applicability of the bionic mechanoreceptor array could be extended to the identification of commonly used materials in daily life, including FEP, PTFE, Kapton, PET, PE, rubber, acrylic, paper, Al, nylon, and others, as illustrated in Fig. 4 (D and E). Distinct triboelectric electric outputs were generated upon contact or separation of the sensor array from each material, exhibiting unique waveforms and relative amplitude characteristics. Furthermore, through an analysis of the interplay between the four response electric signals of the bionic mechanoreceptor array during contact with each material and the correlation among multiple electric signals across different materials, we derived output voltage signals for 10 materials and normalized the voltage signals for each sensor individually. It is evident that these materials occupy varying positions in the frictional electrification series (Fig. 4, F and G).

### Identification system enhanced by machine learning

**Fig. 5.**
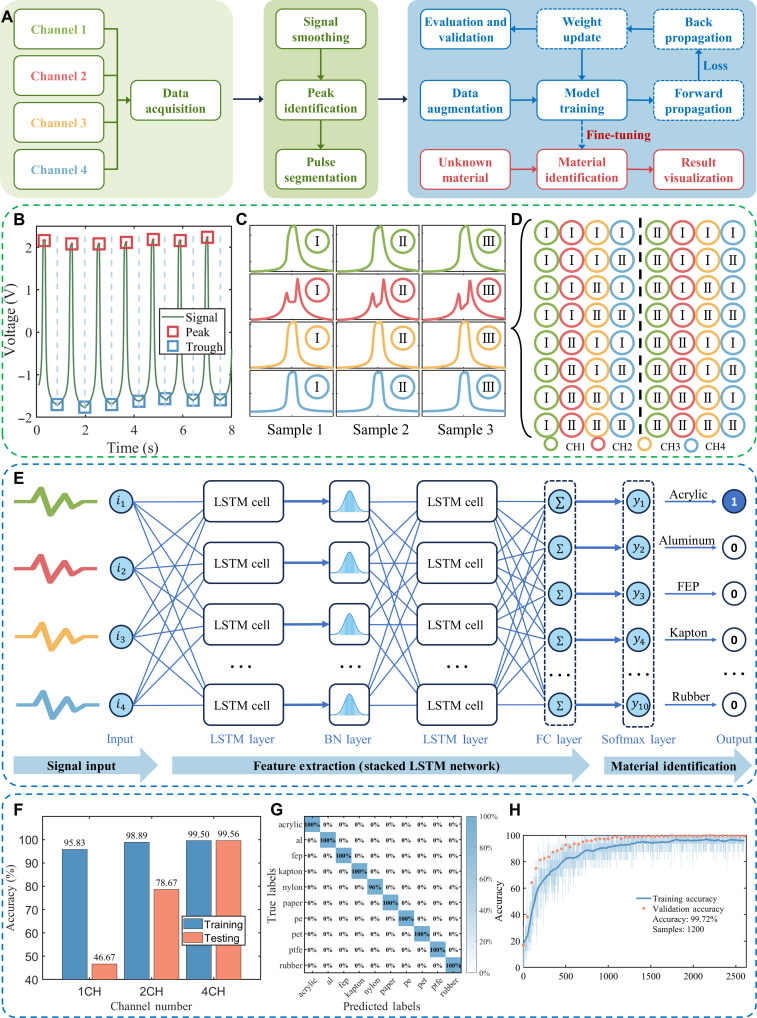
Machine learning–based data processing for high-accuracy material-type identification. (**A**) The overall computational flowchart of machine learning for material identification. (**B**) Outcomes from pulse identification. (**C**) Pulse segmentation for individual signal analysis. (**D**) Expanded sample set through data augmentation. (**E**) Stacked LSTM network architecture with signal input flow. (**F**) Accuracy improvement of the neural network with increased channel input. (**G**) Confusion map of the machine learning results for 10 categories with four channels. (**H**) Training process in fine-tuning.

Machine learning has found extensive applications in processing data with intricate features and variable signal lengths. We used a stacked LSTM network (text S4 and table S3) to handle data collected by a multi-modal sensor array of a biomimetic robotic hand. The model underwent training via supervised learning to recognize unknown materials. The unique advantage of LSTM lies in its capability to process sequences of varying lengths, enabling the model to adapt flexibly to datasets containing sequences of different lengths. Once trained, the model adeptly handled data of various lengths. The overall computational process is depicted in Fig. 5A. Initially, we preprocessed the material data collected by sensors, involving segmenting continuous signals and implementing data augmentation strategies (figs. S31 and S32). Data augmentation effectively expanded the dataset, thereby enhancing training effectiveness. The augmented data were used to train the neural network, and through multiple iterations, the model’s predictions gradually converged to the actual values. The model, trained with a large dataset, was saved as a pretrained model. In the model deployment and inference phase, to adapt the algorithm to different environmental material characteristics, we improved material identification accuracy in various environments through domain-specific fine-tuning. Online training of the pretrained model using a small subset of labeled data from the different environment was conducted in this round of training, requiring fewer samples and being faster. Upon completion of training, our architecture completed an inference in 0.041 s using an AMD Ryzen 7 4800H CPU processor, demonstrating its efficient real-time processing capability. As illustrated in Fig. 5B, during the signal preprocessing stage, the algorithm automatically detected and identified the peaks and troughs of continuous pulse signals (figs. S33 to S42 showed the identification process for peaks and troughs of different waveforms).

On the basis of these peak and trough signals, we segmented multiple continuous pulse signals, as depicted in Fig. 5C. After segmenting the continuous signals captured by sensors, we obtained *n* samples, each representing a pulse. Next, we expanded the number of samples available for training through data augmentation. As illustrated in Fig. 5D, by recombining the segmented *n* samples, we could potentially expand the sample size to *n*^4^. Here, we generated a total of 2000 data entries for 10 categories through data augmentation, allocating 70% for training and 30% for validation. The test dataset did not undergo data augmentation. The overall architecture of the neural network used is shown in Fig. 5E. Stacked LSTM architecture contains two LSTM layers with 16 units each. The detailed structure of the LSTM units is thoroughly described in fig. S43 and text S5. Experimental results, as depicted in Fig. 5F, compare the performance of the neural network trained with single-channel, dual-channel, and quad-channel data. The results showed that the test accuracy of the neural network increased from 46.67% with a single channel to 99.56% with four channels, indicating that increasing the number of signal channels substantially improves identification precision. The confusion matrix of the pretrained model, with an overall accuracy rate of 99.56%, is displayed in Fig. 5G, with only one sample being misclassified. The training process undertaken during the fine-tuning phase of the model is illustrated in fig. S44 and text S6. To ensure the broad applicability of our results, further experiments were conducted to test the performance of the bionic mechanoreceptors in harsh environments. These included high humidity and noisy conditions. In addition, to prevent overfitting, dropout layers were introduced during fine-tuning. The results indicated that the model can achieve 99.72% accuracy in tactile perception training (Fig. 5H).

### The machine learning–assisted somatosensory system

**Fig. 6.**
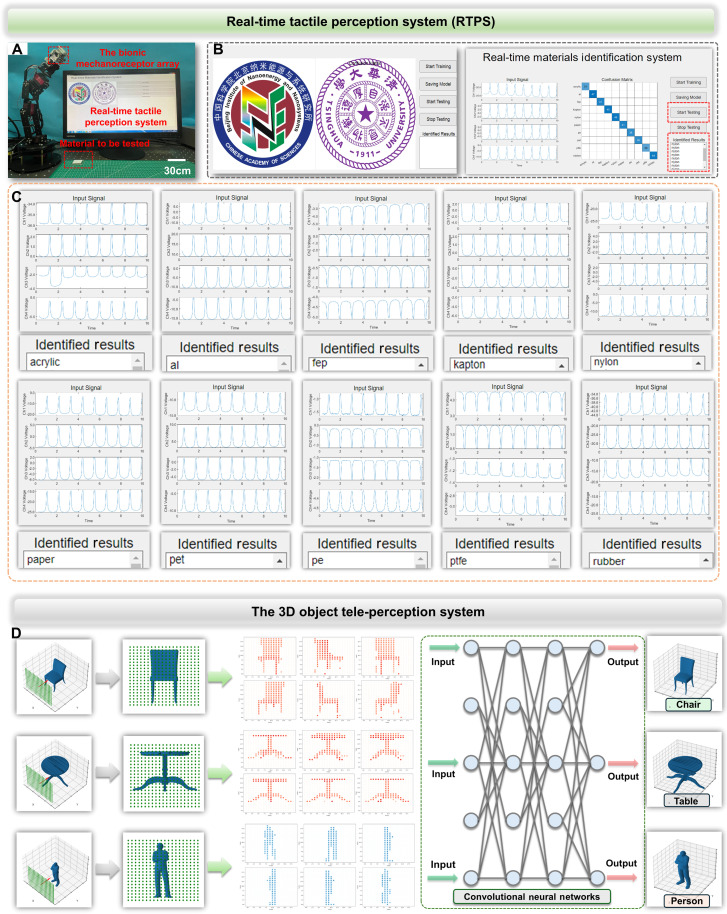
Applications of the multi-receptor skin in tele-perception and tactile perception. (**A**) A photo of a robotic arm with the bionic mechanoreceptor array interacting RTPS. (**B**) Homepage of RTPS. (**C**) The output signal curve and identification results of RTPS when identifying material. (**D**) The 3D object tele-perception system based on the bionic electroreceptor matrix (20 × 20 units) and the CNN.

To interact effectively with the real world, the bionic mechanoreceptor array should have a similar ability to human skin to infer the material type of an object in contact. However, identifying an object’s material type poses a considerable challenge for human skin, especially when dealing with objects that have similar surfaces. By combining the bionic mechanoreceptor array with deep learning–assisted material identification systems, we can identify the material type of common objects in real time, showcasing capabilities beyond human skin perception. To validate the practical feasibility of the bionic mechanoreceptor array in material-type identification, we developed a real-time tactile perception system (RTPS), which integrates deep learning algorithms for immediate material identification. LSTM algorithm excels at processing time-series data, effectively capturing the time-dependent features of tactile signals to enhance accuracy and sensitivity in tactile perception. To better demonstrate the material identification functionality of the RTPS in real-world scenarios, we integrated the bionic mechanoreceptor array into a robotic arm. Figure 6A presents a photograph of a robotic arm with an attached bionic mechanoreceptor array and RTPS, ready to execute a touch command. The RTPS can display real-time parameters such as the raw triboelectric electric signal curve and peak values, saving the signal curves in data file format to a specified folder. Subsequently, these signal curves were input into a pretrained deep learning model, and inference results were obtained (Fig. 6B). During operation, as the robotic arm drove the bionic mechanoreceptor array to touch materials consecutively four times, the acquired signals underwent training (fig. S45). Upon contact between the bionic mechanoreceptor and an object, the RTPS was capable of analyzing the sensed data in real time and rapidly matching it with the data in the database. This guaranteed that the system was both efficient and reliable in practice. Furthermore, the RTPS system was highly scalable. The database could be expanded at any time, allowing for continuous improvement in the system’s identification capabilities. To demonstrate the effectiveness and reliability of the RTPS system in multi-object identification tasks, 10 additional materials were included in RTPS. Even in the case of objects with electrostatic properties, the system was able to successfully identify and distinguish them with high accuracy through multiple training sessions and database optimizations. The final material-type identification results were displayed on the computer terminal interface (Fig. 6C and fig. S46). In addition, the tactile perception process is detailed in movie S3.

Tele-perception refers to the capacity to perceive and comprehend objects, environments, or events beyond conventional perception ranges. The structured doping of inorganic nonmetal nanoparticles in our design enhances the electron capture capability by enlarging charge traps, thereby amplifying the tele-perception perception capability. High-resolution devices often demand considerable computational power and intricate processing circuits, despite their enhanced target discernment capabilities. In such cases, machine learning algorithms offer an alternative approach to augment functionalities while minimizing sensor requirements. A 3D identification artificial tele-perception somatosensory system was developed by inputting scanning results from our bionic electroreceptor perception matrix, which scanned real objects, into a CNN. CNN algorithms excel in processing spatial features, extracting valuable spatial information from multi-dimensional data to improve the accuracy and stability of tele-perception. As demonstrated in Fig. 6D, we increased the number of units in the bionic electroreceptor perception array to 400 (20 × 20) to achieve a more precise electric potential distribution. In addition, by using the “data-to-color” method, the electric potential distribution of the electromagnetic sensors in the simulation results was transformed into 2D images, making it easier for the CNN to extract image features. A 3D model simulation system was built based on the Möller-Trumbore algorithm and the Octree algorithm. This system allowed us to simulate the sensor array’s perception process of different objects at varying distances. The Möller-Trumbore algorithm was used for ray-triangle intersection detection, while the Octree algorithm accelerated the simulation speed. The simulated sensor array provided feedback signals that included the material properties of the objects and the distance between the sensors and the object surfaces. In the neural network component, a four-layer CNN was used. The first two layers were convolutional layers, and the subsequent two were fully connected layers. The rectified linear unit function served as the activation function, and the Adam optimizer was used to expedite the training process. The results of the simulation system indicated that a machine learning–based sensor array could effectively recognize and perceive the shapes and materials of various 3D objects and provide 3D visualization. Moreover, the data collected by the simulated bionic electroreceptor array from different angles (fig. S47) closely matched real-world conditions, laying a solid foundation for the development of future practical devices.

Here, the deep learning CNN selected three common indoor geometric configurations: human, chair, and table. We constructed training and test datasets to perform the 3D identification task based on this system. The training dataset consisted of 300 samples divided into three classes. Each sample in each class had a unique position and angle. The test dataset consisted of three images that were never presented to the training dataset. Initially, we constructed a four-channel miniature CNN because our sensory data contained rich and salient object features, allowing us to use fewer convolutional kernels to achieve more accurate identification results. At the start of deep learning, identification accuracy reached 100% after three training cycles (fig. S48). The trained 3D identification system was then tested on the dataset, with results as shown in the figure. The categories “chair,” “human,” and “table” were labeled “0,” “1,” and “2,” respectively. If an image of a person was input into the system, the output would be 1, indicating successful identification of a person. Similar results were obtained when images of chairs and tables were input into the system. In addition, using machine learning algorithms and deep learning models, we can train the system to learn the color characteristics specific to different materials from spectral data and correlate them with the corresponding material types. In this way, the system can accurately identify the material of objects even over long distances or in invisible conditions, opening more opportunities for applications in areas such as smart robotics, autonomous vehicles, and industrial manufacturing. The core and profound significance of tele-perception lies in the expansion of human perceptual capabilities, our tactile, visual, auditory, and other sensory abilities.

## DISCUSSION

Our investigation unveils the use of tele-perception as a means to unlock innovative dimensions of human perception and cognition, thereby surpassing the constraints of noncontact sensors and traditional sensory modalities. Our designed bio-inspired multi-receptor skin achieves superior tele-perception and tactile perception through structured incorporation of inorganic nonmetal nanoparticles and integration with advanced deep learning algorithms. On the basis of experimental observations and COMSOL simulations, we propose a charge trap mechanism initiated by induced polarization, facilitated by the structured doping of inorganic nonmetal nanoparticles. This mechanism augments tele-perception somatosensation and sets a record-high sensitivity. This strategic shift effectively addresses the prior challenge of disorganized doping of inorganic nonmetal nanoparticles within elastic substrates, mitigating confusion in the direction of the local electric fields (*61*). Furthermore, we demonstrate that our enhanced adaptive pulse identification, facilitated by the LSTM algorithm, further elevates the tactile perception for material identification, achieving an impressive accuracy of 99.56% alongside accelerated processing speeds. Compared to the intricate process that humans must undergo to integrate multiple senses (such as sight and touch) for tactile perception, our tactile perception system exhibited highly efficient material identification for real applications. In addition, we have represented the feasibility of incorporation of data derived from real object scans using a 2D sensor matrix into a CNN, thereby facilitating the development of a multi-receptor skin with both tele-perception somatosensation and material identification. In contrast, traditional 3D identification via charge-coupled device and infrared cameras entails more complex circuits and higher energy consumption. These distinctive advantages render our multi-receptor skin more competitive in certain applications, including the following: (i) independence from external power sources; (ii) high level of signal stability; (iii) highly sensitive characteristics; (iv) our proposed concept of tele-perception surpasses the limitations of traditional noncontact sensors in terms of operating distance and sensing modes; and (v) the design incorporates a doped elastomer film treated with high-voltage polarization, enabling multi-receptor skin to accurately detect changes in electrical signals from a distance without direct contact with the object’s surface. By integrating multiple sensory inputs, the multi-receptor skin enhances human perception in HMIs and humanoid robotics, akin to a sixth sense. Looking forward, intelligent fingertip interfaces with robots or even the human body hold promise for advancing human-machine interaction and sensory immersion, facilitating more sophisticated and practical application scenarios.

## MATERIALS AND METHODS

### Materials

Silicon wafers, PDMS, curing agent, PTFE emulsion, SiO_2_, TiO_2_, BaTiO_3_, and SrTiO_3_ powders were all commercially sourced and used without further purification.

### Fabrication of silicon wafer templates

Initially, a layer of photoresist was applied to silicon wafer substrates. Selective exposure of the photoresist was achieved through ultraviolet (UV) exposure using a mask. Subsequently, the exposed areas were dissolved using a developer solution, revealing the pattern from the mask on the photoresist. A selective etching process was carried out on the substrate using inductively coupled plasma etching. The remaining photoresist protected the substrate from alteration during these processes. After etching, the photoresist was removed using *N*-methyl-pyrrolidone to obtain microstructured templates.

### Preparation of PTFE & PDMS composite film

Using optimal parameters as an example: Initially, 0.1 ml of PTFE emulsion and 1 ml of PDMS (optimal parameters) were uniformly mixed, with the addition of 0.5 ml of *n*-hexane to reduce viscosity. The mixture was stirred at 500 rpm on a magnetic stirrer for 2 hours at 80°C to remove water from the PTFE emulsion. After cooling to room temperature, the curing agent (Dow Corning) was added in a 1:10 ratio (curing agent: PDMS). Subsequently, the mixture was centrifuged at 1000 rpm for 5 min to eliminate air bubbles. After degassing, the resulting mixture was spin-coated onto glass and silicon templates with microstructures, followed by curing at 100°C for 1 hour. Last, the PDMS-PTFE composite film was peeled off from the substrate.

### Structured doping process of inorganic nonmetal nanoparticles

Using optimal parameters as an example: (i) Weigh 0.12 g of SrTiO_3_ inorganic nonmetal nanoparticles. (ii) Mix SrTiO_3_ inorganic nonmetal nanoparticles with 10 ml of ethanol and ultrasonicate for 20 min to form a homogeneous suspension. (iii) Add 1 ml of PDMS precursor to the SrTiO_3_-ethanol suspension and stir for 10 min. (iv) Place the mixture in an oven at 90°C for more than 10 hours to ensure complete evaporation of ethanol. (v) Add the curing agent and stir the mixture for 5 min. (vi) Pour the mixture of SrTiO_3_ inorganic nonmetal nanoparticles and PDMS onto the microstructured PDMS-PTFE composite film. (vii) Cure the composite material in an oven at 80°C for 3 hours. Thus, a composite film with structured doping of inorganic nonmetal nanoparticles was prepared.

### Preparation of the AgNW electrode

PDMS with a weight ratio of 10:1 of elastomer base to curing agent was spin-coated on an acrylic substrate and cured at 70°C for 2 hours. After plasma treatment, the AgNW solution was spin-coated on the pretreated PDMS substrate, resulting in the AgNW/PDMS transparent conductive film after solvent evaporation for 1 hour.

### Manufacture of the multi-receptor skin

Applying the template method and bonding technology, a structured nanocomposite array-based multi-receptor skin can be fabricated by bonding a PDMS-PTFE composite membrane subjected to plasma treatment, a PDMS-PTFE composite film with structured doping of inorganic nonmetal nanoparticles, and a AgNW/PDMS conductive film.

### Electric measurement and characterization

A linear motor (LinMotS01-72/500) was used to control the distance between objects and the artificial sensor. The SEM images were obtained using a scanning electron microscope (Hitachi SU8020). Plasma cleaner provided voltage for the precharging process. Open-circuit voltage was measured using a programmable electrostatic voltmeter (Keithley 6514). For basic output performance testing of the bio-inspired artificial sensor, a programmable electrostatic voltmeter (Keithley 6514) was directly connected to a synchronized data acquisition card (National Instruments 6346) to measure multi-channel voltage signals. A robotic arm with the artificial sensor was used for executing touch commands. A multi-channel data acquisition program developed on the LabVIEW platform was used for data collection, processing, and storage.

## Supplementary Materials

### This PDF file includes:

Supplementary Text S1 to S6 Figs. S1 to S48 Tables S1 to S3 Legends for movies S1 to S3 References
