# Supplementary material for: Multi-receptor skin with highly sensitive tele-perception somatosensory
Source: Sci Adv. 2024 Sep 11;10(37):eadp8681. doi: 10.1126/sciadv.adp8681 (PMC11389779; doi:10.1126/sciadv.adp8681)
Supplement: Supplementary file 1 — Supplementary Text S1 to S6 Figs. S1 to S48 Tables S1 to S3 Legends for movies S1 to S3 References [file sciadv.adp8681_sm.pdf]

Science Advances
AAAS

Supplementary Materials for

Multi-receptor skin with highly sensitive tele-perception somatosensory

Yan Du *et al.*

Corresponding author: Tianling Ren, rentl@tsinghua.edu.cn; Daping Chu, dpc31@cam.ac.uk; Zhonglin Wang, zhong.wang@mse.gatech.edu; Di Wei, dw344@cam.ac.uk

Sci. Adv. **10**, eadp8681 (2024)
DOI: 10.1126/sciadv.adp8681

The PDF file includes:

Supplementary Text S1 to S6
Figs. S1 to S48
Tables S1 to S3
Legends for movies S1 to S3
References

Other Supplementary Material for this manuscript includes the following:

Movies S1 to S3

Supplementary Text

Supplementary Text 1

The JAVA algorithm for randomly generating cavity size.

```
int NUMBER_OF_Holes = 10;
int ind = 0;
double hx, hy, hr, hz = 0.0;
double zone_width = 1.0;
double zone_height = 0.5;
double HOLE_MIN_RADIUS = 0.01;
double HOLE_MAX_RADIUS = 0.1;
model.component("comp1").geom("geom1").lengthUnit("m");
model.component("comp1").geom("geom1").selection().create("csel1", "CumulativeSelection");
  while (ind < NUMBER_OF_Holes) {
    hx = Math.random()*zone_width;
    hy = Math.random()*zone_height;
    hr = Math.random()*(HOLE_MAX_RADIUS-
HOLE_MIN_RADIUS)+HOLE_MIN_RADIUS;
    hz = Math.random()*(HOLE_MAX_RADIUS-
HOLE_MIN_RADIUS)+HOLE_MIN_RADIUS;
if ((hx+hr) > (zone_width)) {continue; }
if ((hy+hz) > (zone_height)) {continue; }
if ((hx-hr) < 0) {continue; }
if ((hy-hz) < 0) {continue; }
model.component("comp1").geom("geom1").create("e"+ind, "Ellipse");
model.component("comp1").geom("geom1").feature("e"+ind).set("a", hr);
model.component("comp1").geom("geom1").feature("e"+ind).set("b", hz);
model.component("comp1").geom("geom1").feature("e"+ind).set("pos", new double[]{hx, hy});
model.component("comp1").geom("geom1").feature("e"+ind).set("contributeto", "csel1");
ind++;}
model.component("comp1").geom("geom1").create("r1", "Rectangle");
```

```
model.component("comp1").geom("geom1").feature("r1").set("size", new double[]{zone_width, zone_height});
model.component("comp1").geom("geom1").create("dif1", "Difference");
model.component("comp1").geom("geom1").feature("dif1").selection("input").set("r1");
model.component("comp1").geom("geom1").feature("dif1").selection("input2").named("csel1");
model.component("comp1").geom("geom1").feature("dif1").set("keepsubtract", true);
model.component("comp1").geom("geom1").create("dif2", "Difference");
model.component("comp1").geom("geom1").feature("dif2").selection("input").set("dif1");
model.component("comp1").geom("geom1").feature("dif2").selection("input2").named("csel2");
model.component("comp1").geom("geom1").feature("dif2").set("keepsubtract", true);
model.component("comp1").geom("geom1").run();
```

Supplementary Text 2

The JAVA algorithm randomly generates the number of particles in the cavity.

```
int NUMBER_OF_particles = 90;
   int ind = 0;
   double hx, hy = 0.0;
   double hr = 0.01;
   double zone_width = 1.0;
   double zone_height = 0.5;
   model.component("comp1").geom("geom1").lengthUnit("m");
   model.component("comp1").geom("geom1").selection().create("csel2", "CumulativeSelection");
   while (ind < NUMBER_OF_particles) {
     hx = Math.random()*zone_width;
     hy = Math.random()*zone_height;
     if ((hx+hr) > (zone_width)) {continue; }
     if ((hy+hr) > (zone_height)) {continue; }
     if ((hx-hr) < 0) {continue; }
     if ((hy-hr) < 0) {continue; }
     model.component("comp1").geom("geom1").create("c"+ind, "Circle");
     model.component("comp1").geom("geom1").feature("c"+ind).set("r", hr);
```

```
model.component("comp1").geom("geom1").feature("c"+ind).set("pos", new double[]{hx, hy});
model.component("comp1").geom("geom1").feature("c"+ind).set("contributeto", "csel2");
ind++; }
```

Supplementary Text 3

Explanation and Step-by-Step Instructions:

1. **Start**: Program initiation.
2. **Initialization**: Initialize system settings and hardware connections.
3. **Begin Loop**: Enter the main loop, continuously monitoring and processing voltage signals.
4. **Read Voltage Signal**: Acquire the current voltage value from the data acquisition device (e.g., NI DAQ).
5. **Compare Threshold**: Compare the acquired voltage value with preset thresholds to determine which light control range the voltage falls into.
6. **Control Light State**:
   - Control the on/off state of the corresponding colored light based on the voltage range.
   - Each light has a specific voltage threshold range; if the voltage is outside this range, the light remains off.
7. **Update Interface**: Real-time update of the LabVIEW user interface to display the current voltage value and the status of the five lights.
8. **Wait for Next Loop**: After a certain delay, proceed to the next iteration of the main loop.
9. **End Loop**: Conclude the loop and exit the program according to the designed termination conditions.

Supplementary Text 4

The detailed settings of the proposed model.

This LSTM network utilizes a sequence input layer to process tactile pulse sequences with dynamic lengths. Following the input layer are two LSTM layers, each with 16 units, and they do not return the full sequence; instead, they output only the last state of the sequence. Each LSTM layer is followed by a batch normalization layer (BN), which standardizes the activation outputs of the previous layer to accelerate training speed and enhance model stability. After the second LSTM layer, there is a dropout layer with a dropout rate of 0.5 to reduce overfitting. The final part of the network

is a fully connected layer (FC) that maps the data to 10 neurons, corresponding to 10 classification categories, and then the outputs are converted into a probability distribution by a Softmax layer, with the classification layer completing the category prediction.

Supplementary Text 5

The data flow within an LSTM unit

In Long Short-Term Memory networks (LSTM), each unit is composed of a Cell State and an Output State, primarily including three gates: the Input Gate, the Forget Gate, and the Output Gate. The functions of these gates are realized through specific computational formulas, with the input being the current time step's input X_t. In these formulas, W represents weight matrices that transform inputs and the previous hidden state, b denotes bias vectors that adjust the outputs of these transformations, and h_{t-1} is the hidden state from the previous time step, carrying forward information from earlier in the sequence.

The Input Gate consists of two parts: a sigmoid layer and a tanh layer. The computational formulas are:

$$i_t = \sigma(W_{xi}X_t + W_{hi}h_{t-1} + b_i)$$

$$\widetilde{C_t} = \tanh(W_{xC}X_t + W_{hC}h_{t-1} + b_C)$$

Here, i_t is the output of the sigmoid layer of the Input Gate, determining how much of the new input information will be added to the cell state. $\widetilde{C_t}$is the output of the tanh layer, providing new candidate values for updating the cell state.

The Forget Gate decides how much of the old information in the cell state is retained through the following formula:

$$f_t = \sigma\left(W_{xf}X_t + W_{hf}h_{t-1} + b_f\right)$$

Here, f_t is the output of the sigmoid layer, deciding which information from the previous unit's cell state C_{t-1} should be forgotten or retained.

Lastly, the role of the Output Gate is to determine which part of the cell state should be output to the next hidden state. Its formula is:

$$o_t = \sigma(W_{xo}X_t + W_{ho}h_{t-1} + b_o)$$

$$h_t = o_t * \tanh(C_t)$$

Here, o_t is the output of the sigmoid layer, deciding which parts of the cell state C_t will be output.

Integrating the functions of these gates, LSTM units effectively control the flow of information: the Input Gate controls the entry of new information, the Forget Gate decides what old information to retain, and the Output Gate controls the information output from the cell state to the next hidden state. These mechanisms collectively enable LSTMs to process and remember long-term sequential dat(a) overcoming the issues of vanishing and exploding gradients found in traditional RNNs.

Supplementary Text 6

The method for fine-tuning the model

In the pre-training phase, an initial 389 samples were collected using a four-channel sensor, and the model was trained with data from 10 known materials. Data augmentation expanded the pre-training dataset to 2000 entries, which improved the model's capacity to learn features from the labeled data. After deploying the model to a new environment, it was fine-tuned with material data from that specific environment. The fine-tuning process does not alter the pre-trained LSTM layers, only the output layer is retrained from scratch to ensure the fine-tuned model's performance. During the training phase, the output layer, including the fully connected and softmax layers, underwent random initialization, with the number of output nodes matching the number of material categories to be learned. Due to the limited data collected in the new environment, the same data augmentation techniques as in the pre-training phase were utilized to enlarge the dataset. In terms of results, fine-tuning achieved good results with very low cost. We collected a total of 24 data for the 5 samples that needed to be identified in the new environment, which is a very small amount of data. Before fine-tuning, due to changes in the testing environment, the model's accuracy in identifying materials in the new environment was only 30%. However, after fine-tuning, the test accuracy for material classification reached 100%. This result demonstrates the effectiveness of the fine-tuning strategy we adopted.

Supplementary Figures

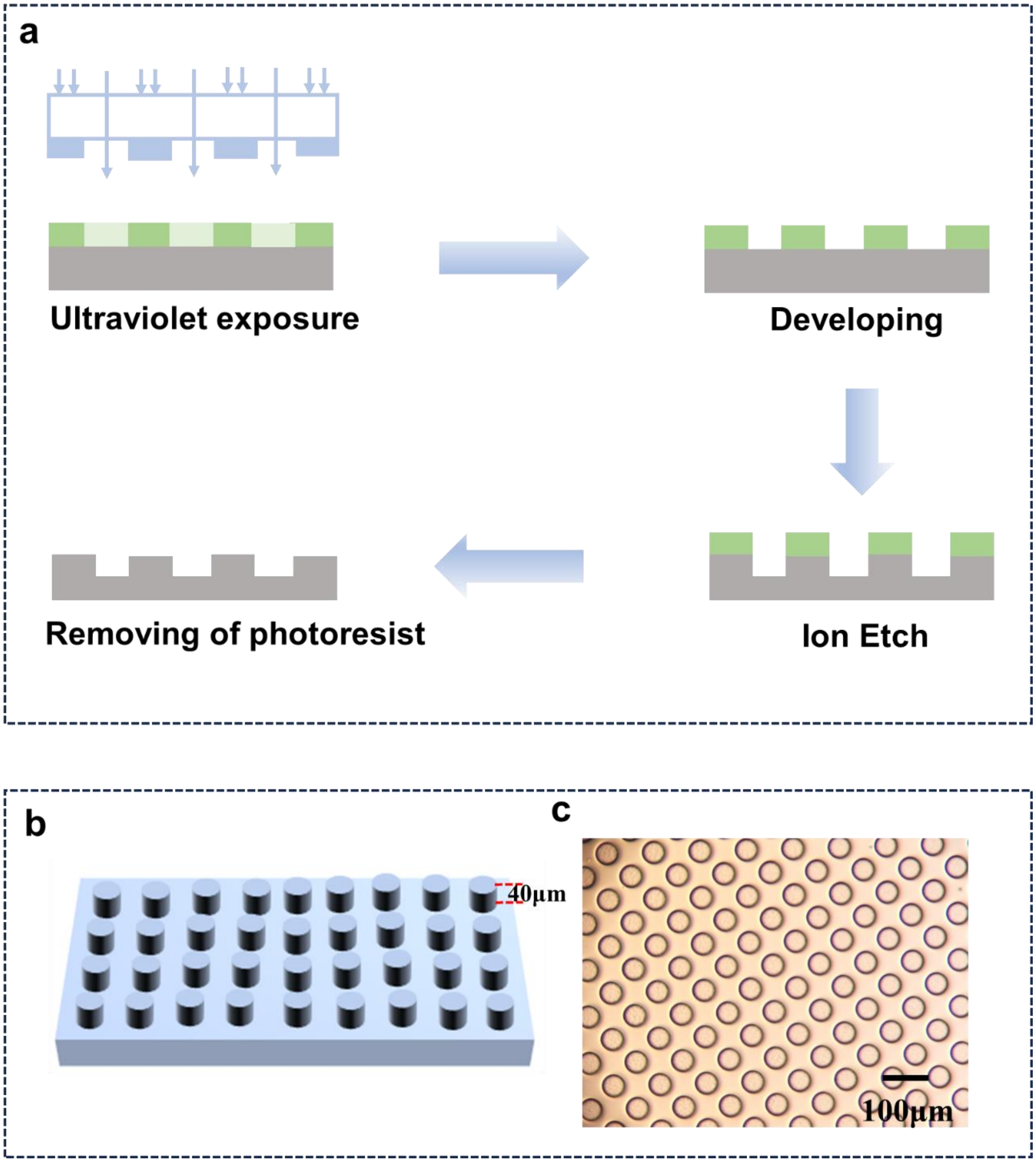

Fig. S1. Construction of microstructured template. (a) Dry etching silicon wafer technology. (b) Three-dimensional model of the surface of the silicon wafer after ion etching. (c) The surface morphology of a silicon wafer after ion etching under an electron microscope.

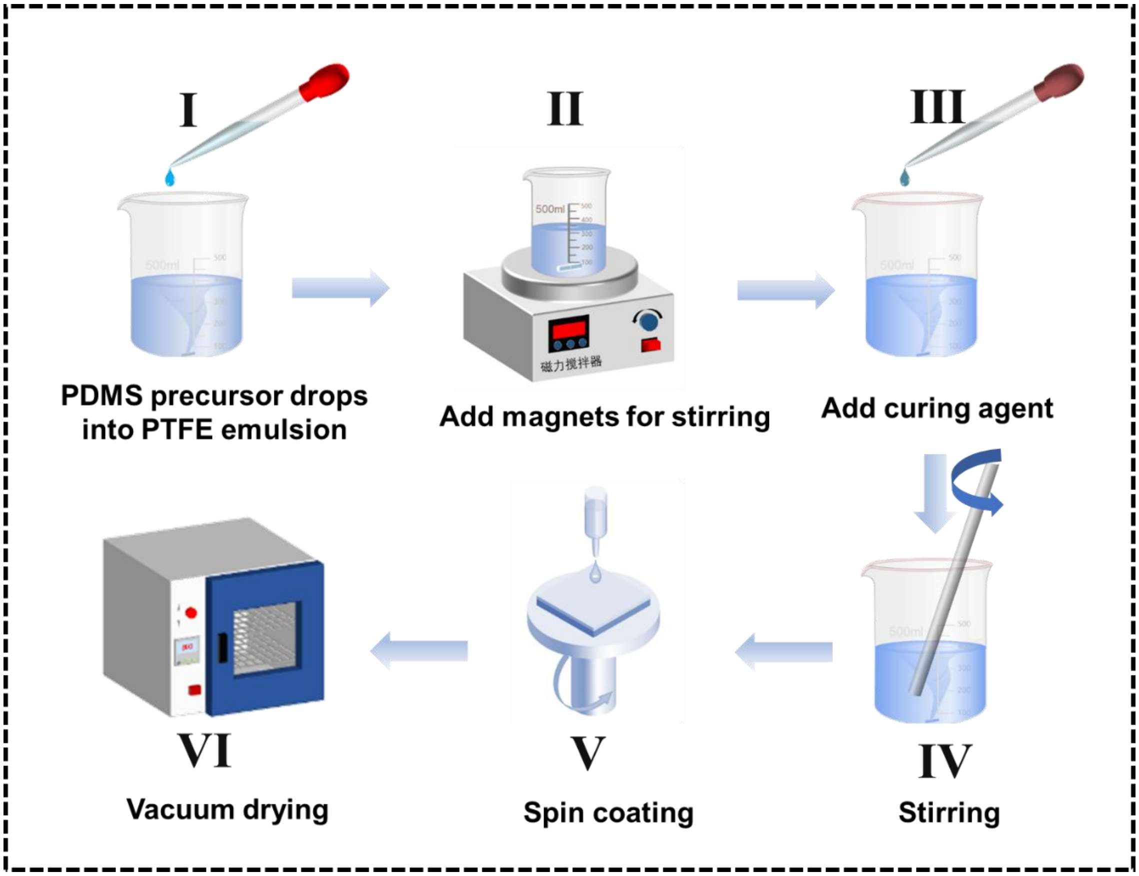

Fig. S2. The manufacturing process of a PDMS film doped with PTFE.

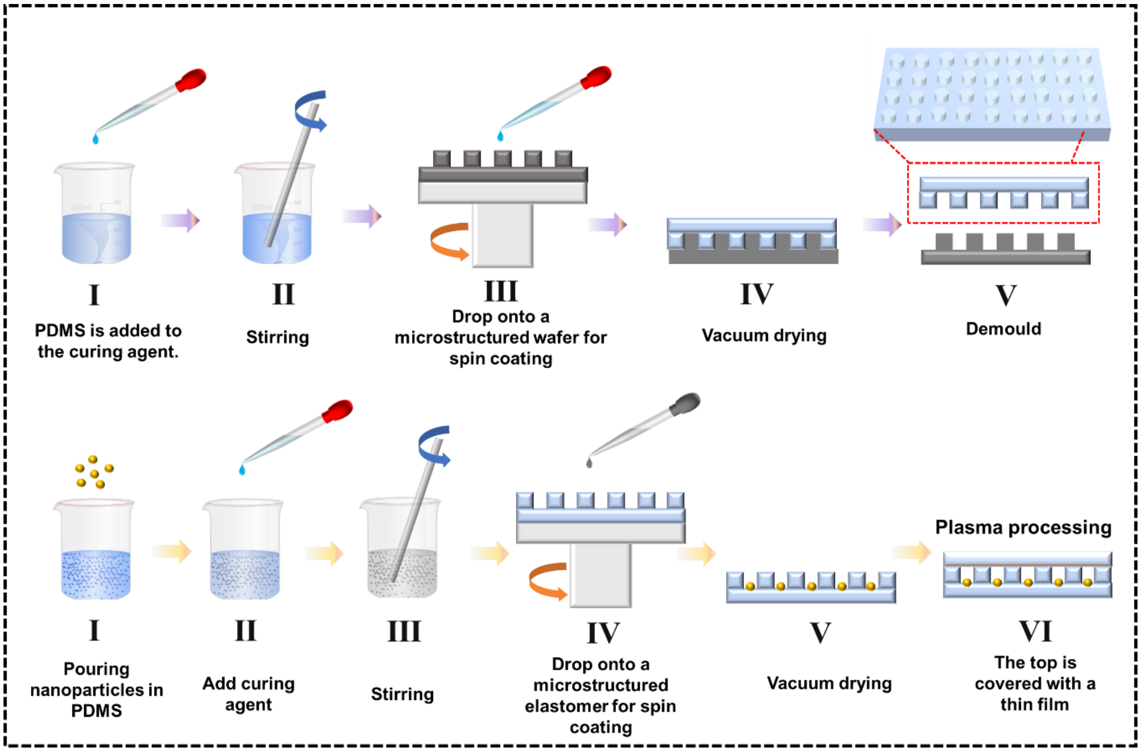

Fig. S3. Preparation technology of elastomer based on the structured doping array of nanoparticles.

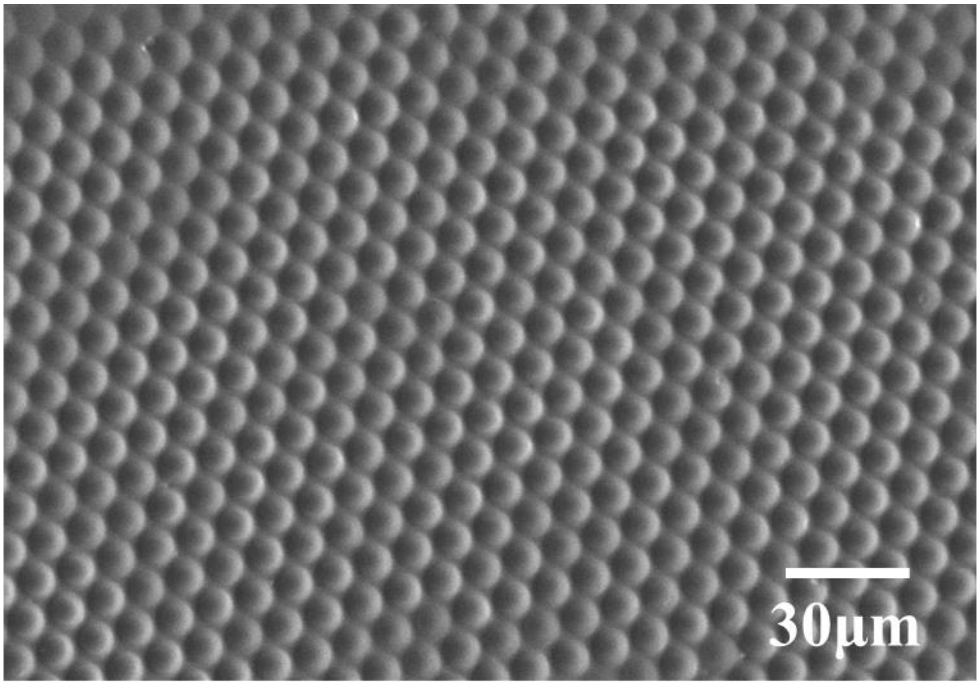

Fig. S4. The surface SEM image of microstructured PDMS (Polydimethylsiloxane) thin film.

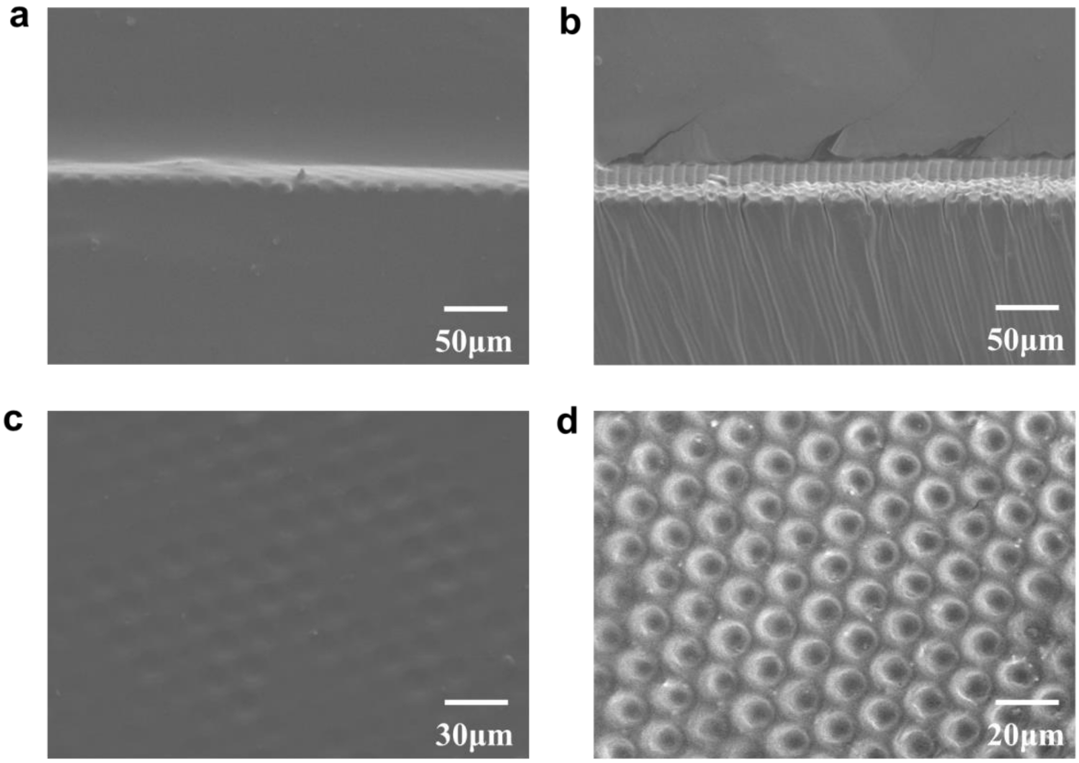

Fig. S5. SEM images of elastomer. (a) SEM cross-sectional image of the surface of a microstructured PDMS (polydimethylsiloxane) film with low-velocity spin-coated $SrTiO_3$ particles. (b) SEM cross-sectional image of the surface of a microstructured PDMS film with high-velocity spin-coated $SrTiO_3$ particles. (c) SEM planar image of the surface of a microstructured PDMS film with low-speed spin-coated $SrTiO_3$ particles. (d) SEM planar image of the surface of microstructured PDMS film with high speed spin-coated $SrTiO_3$particles (Micropore depth:1.6μm).

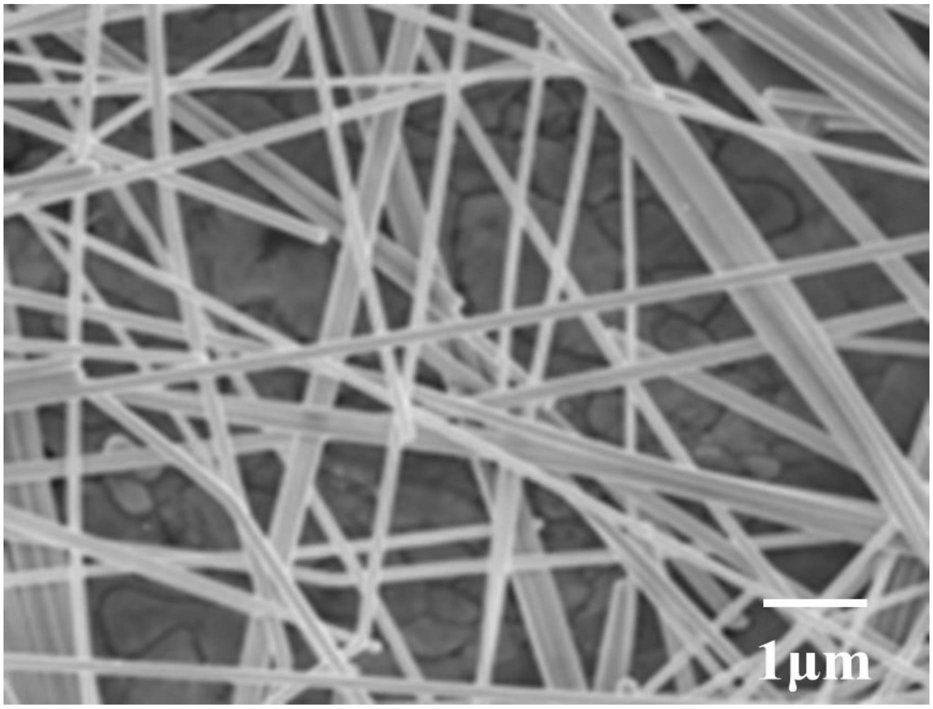

Fig. S6. SEM image of AgNWs (Silver Nanowires).

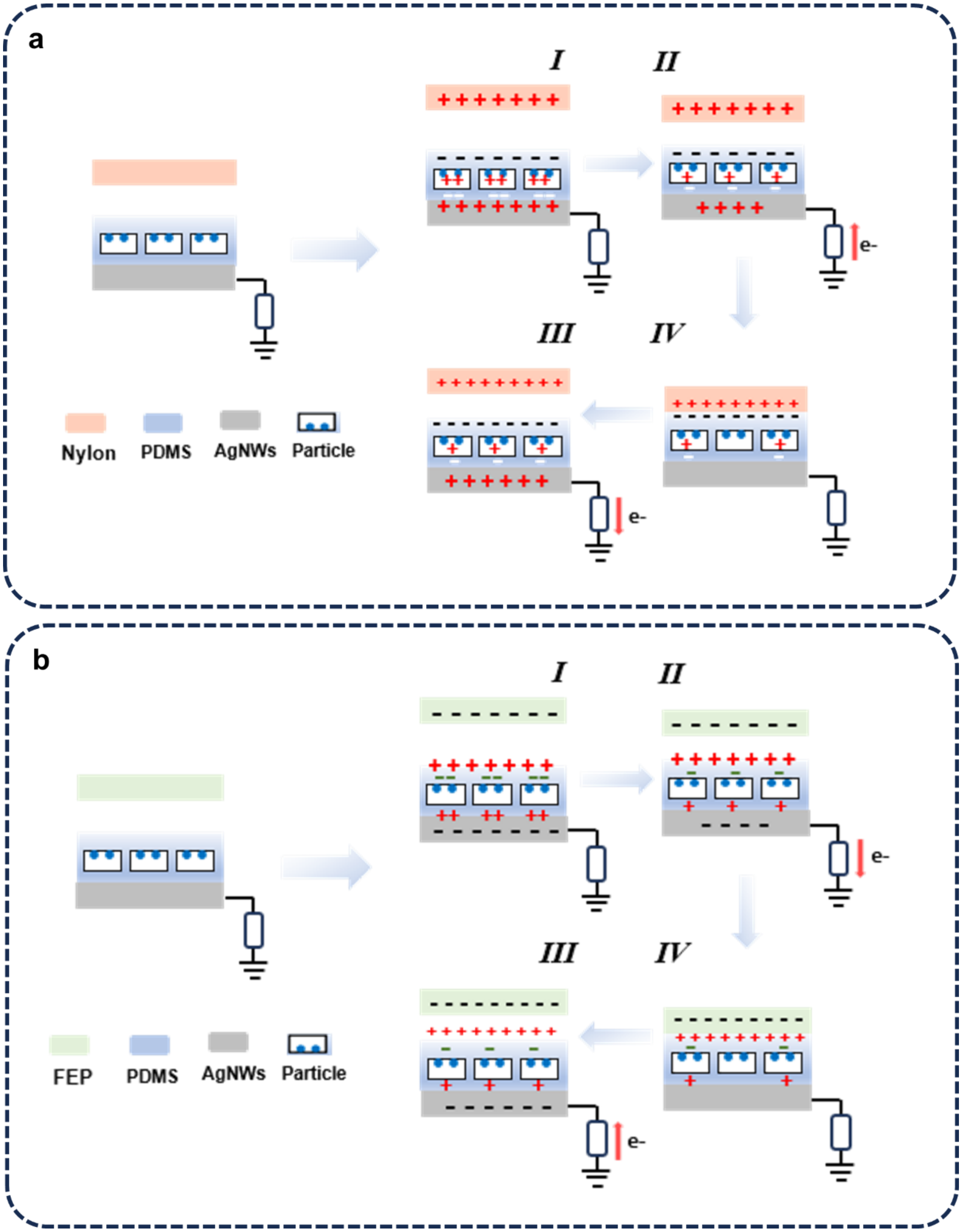

Fig. S7. The specific working principle of the multi-receptor skin. (a) The positively charged objects move closer. **(b)** The negatively charged objects move closer.

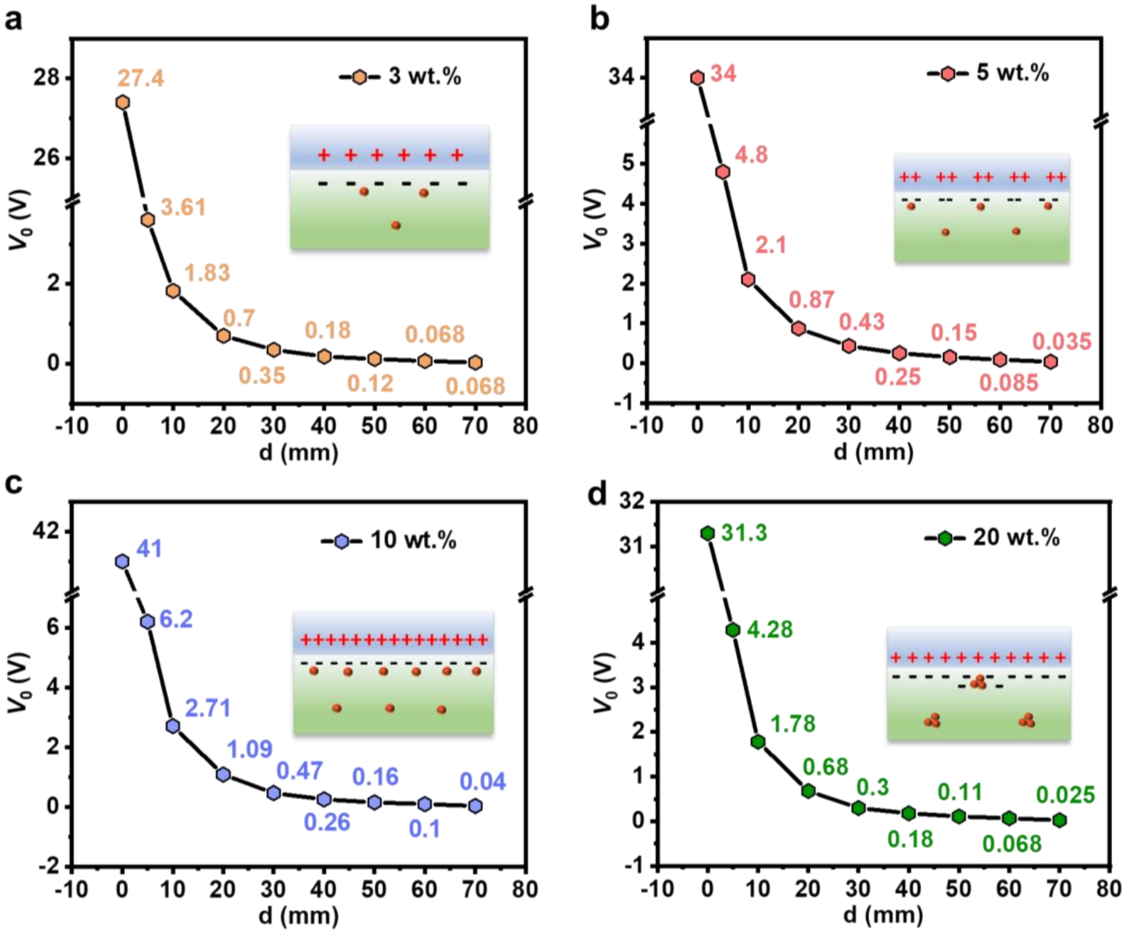

Fig. S8. The output of elastomers synthesized by PDMS doped with PTFE emulsions of different mass fractions. (a) 3%, (b) 5%, (c) 10% and (d) 20%. (The experiment selects nylon as the sensing target close to the output voltage.)

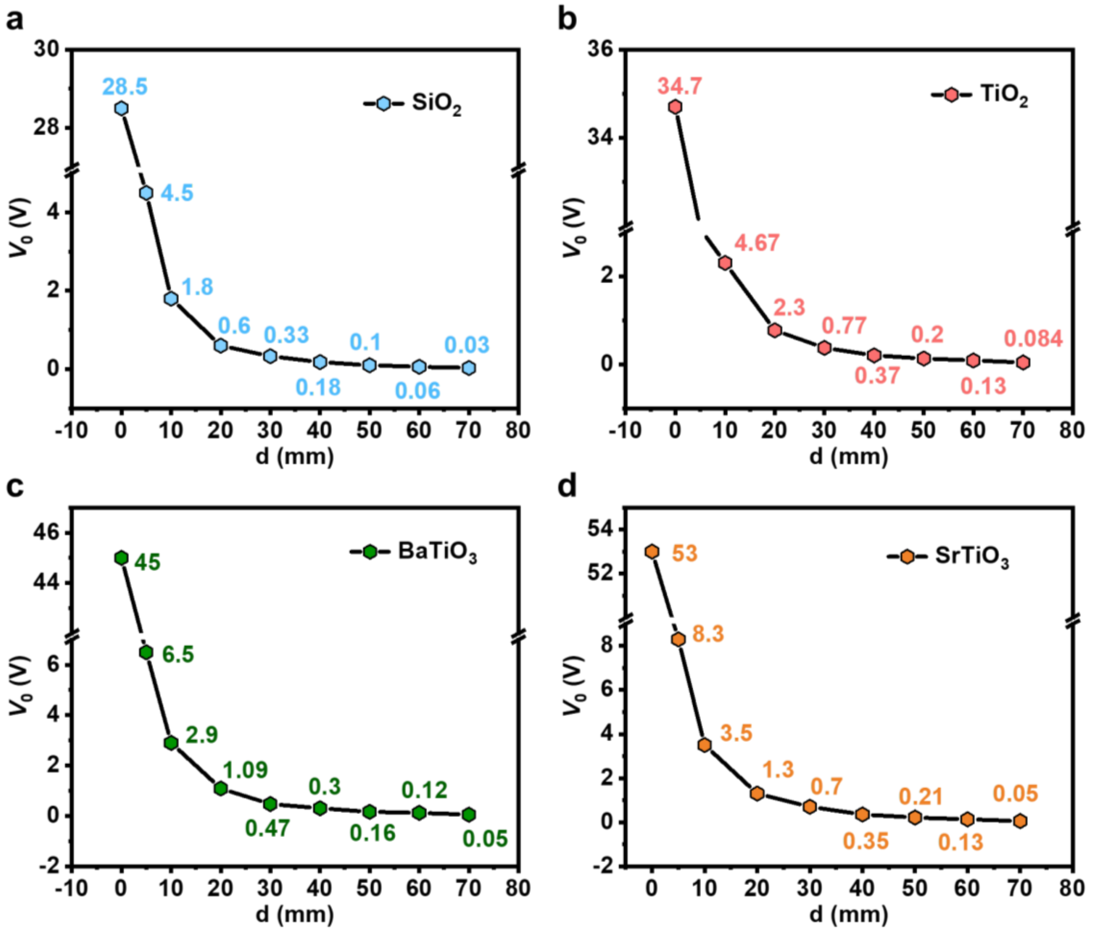

Fig. S9. The output of elastomers synthesized by PDMS doped with different particle. (a) SiO_2, (b) TiO_2, (c) $BaTiO_3$ and (d) $SrTiO_3$. (The experiment selects nylon as the sensing target close to the output voltage.)

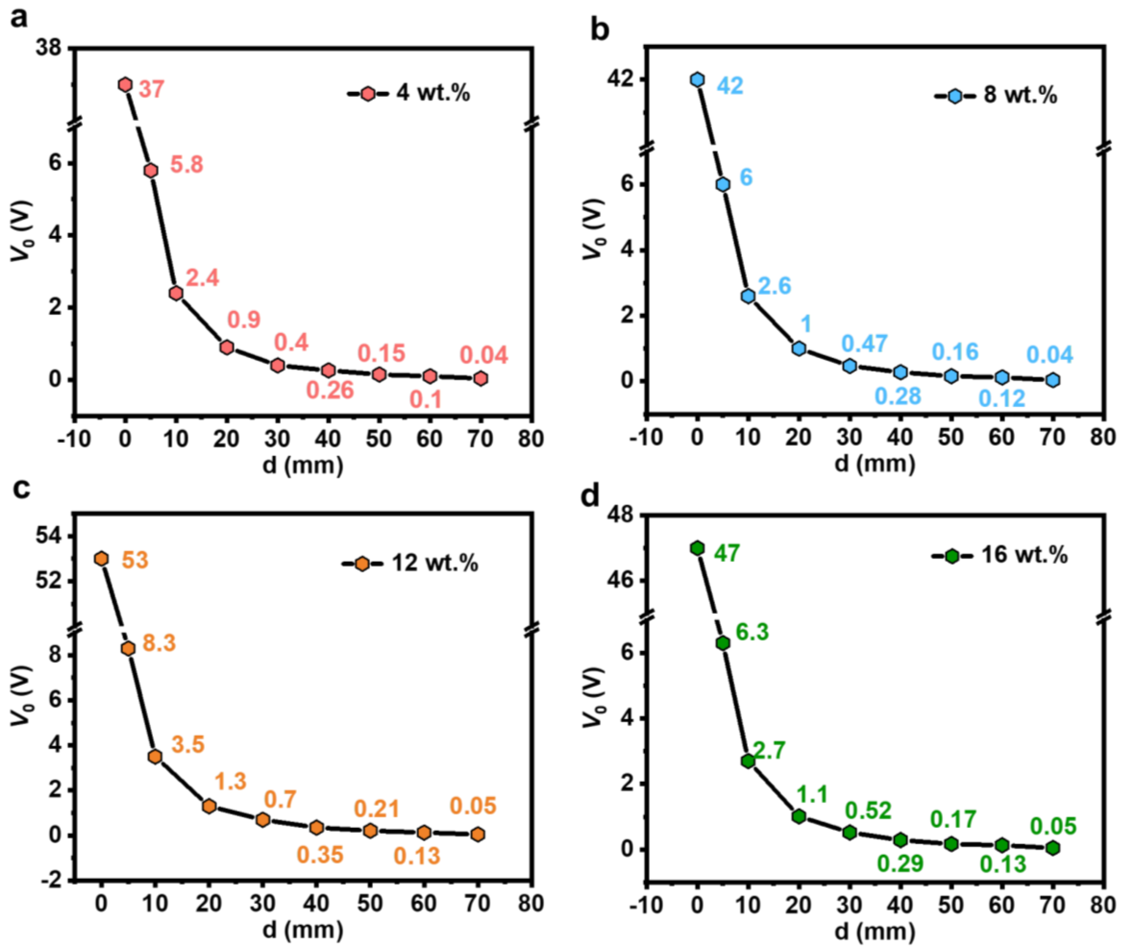

Fig. S10. The output of elastomers synthesized by PDMS doped with $SrTiO_3$ particle of different mass fractions. (a) 4%, (b) 8%, (c) 12% and (d) 16%. (The experiment selects nylon as the sensing target close to the output voltage.)

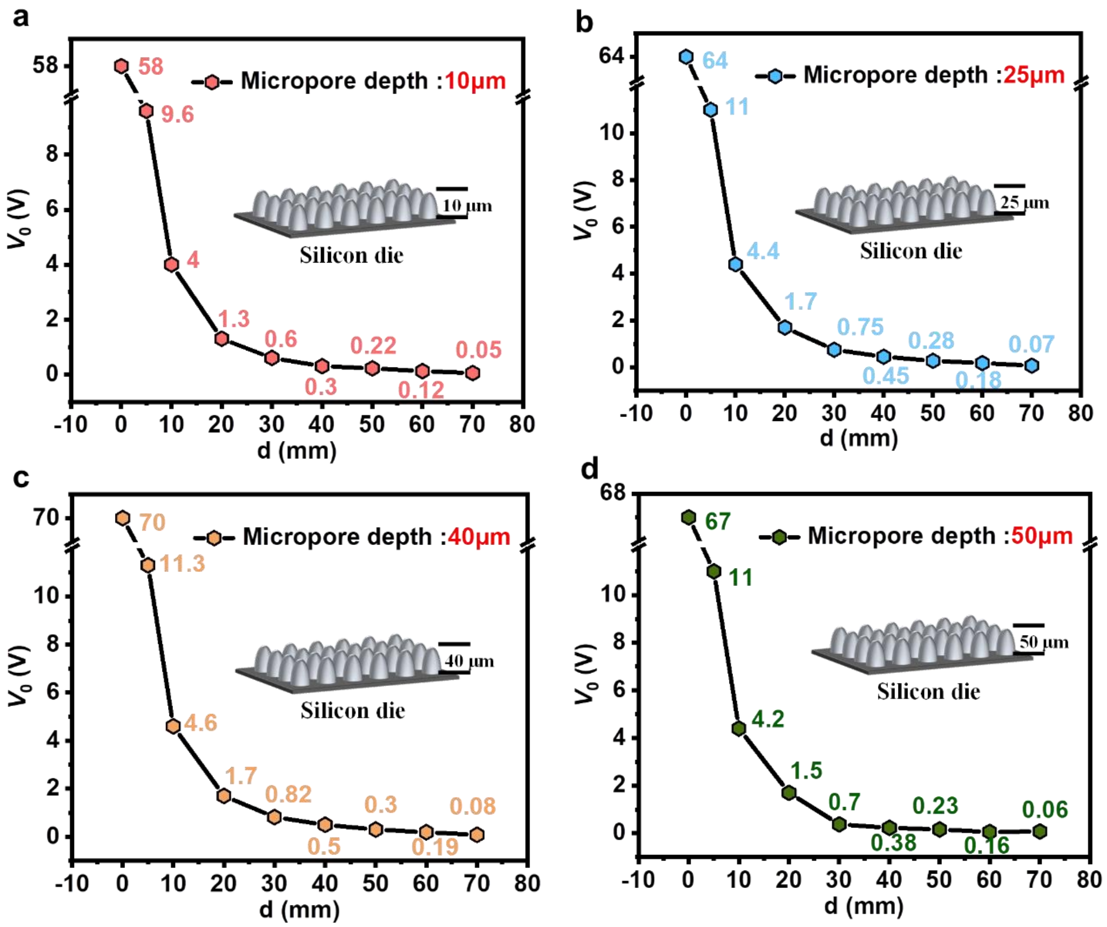

Fig. S11. The output of elastomers synthesized by PDMS doped with with different micropore depth. (a) 10 μm, (b) 25 μm, (c) 40 μm and (d) 50 μm. (The experiment selects nylon as the sensing target close to the output voltage.)

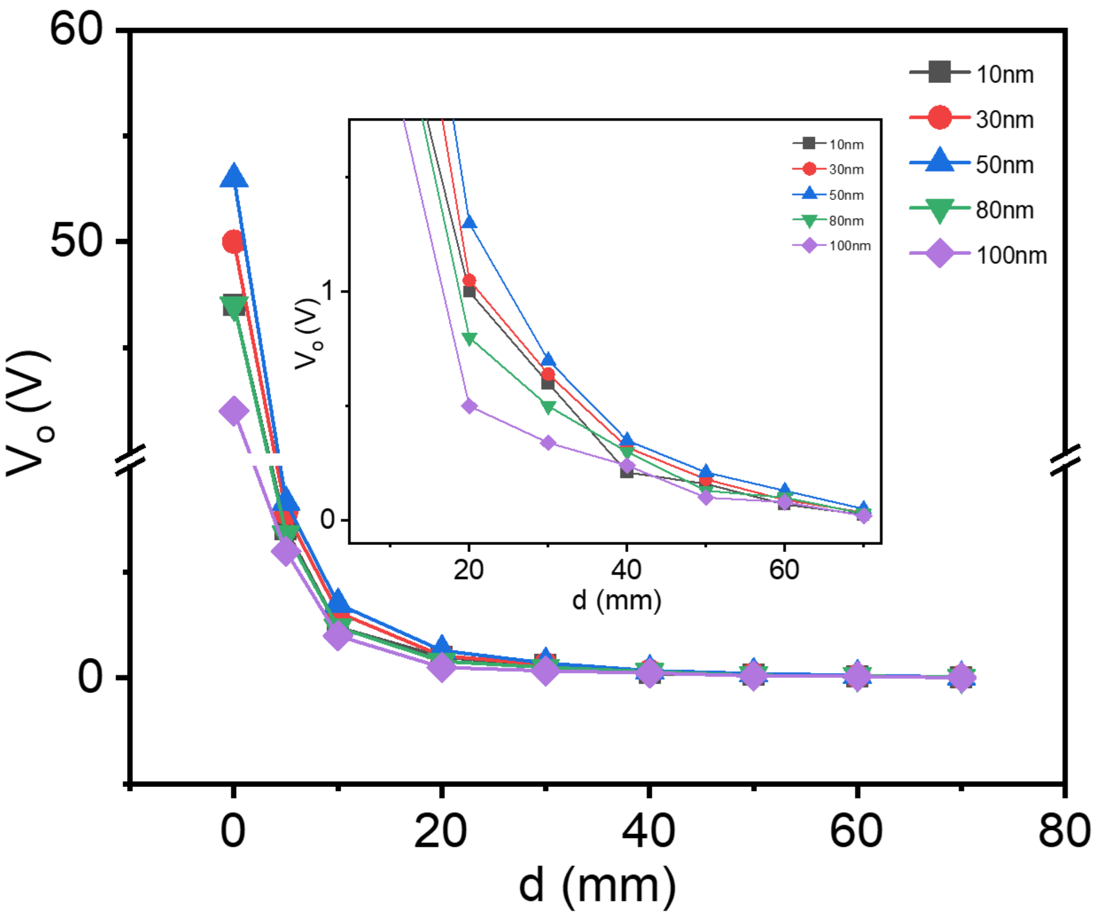

Fig. S12. Output voltage for different $SrTiO_3$ nanoparticle sizes.

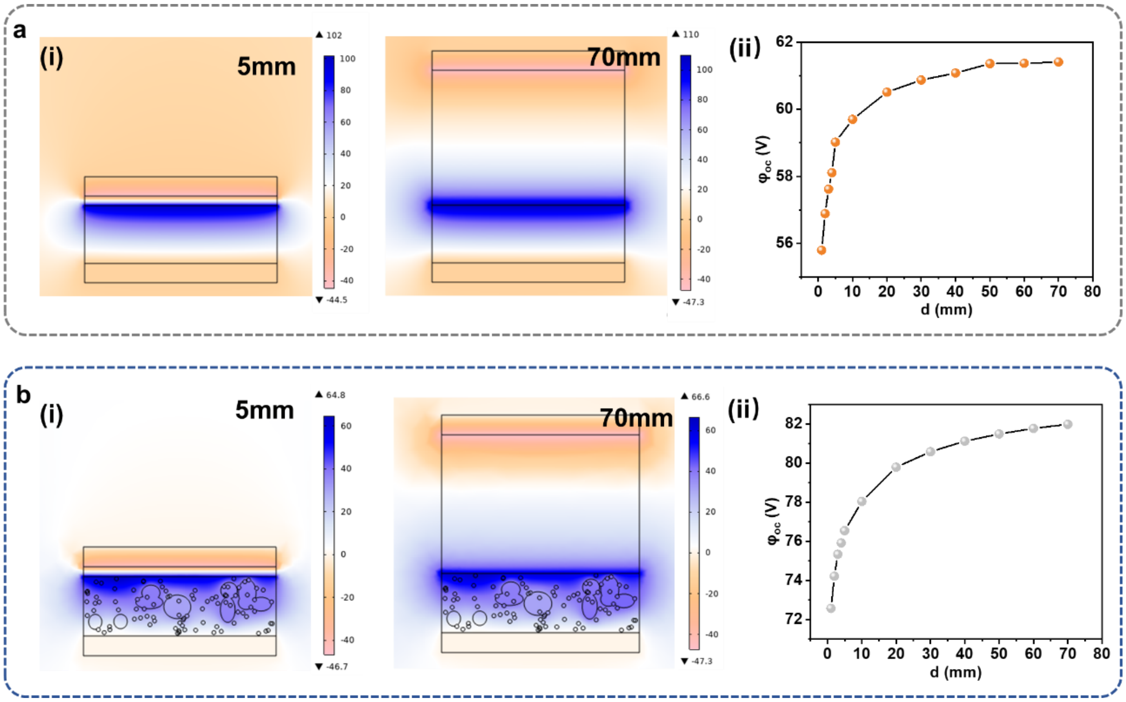

Fig. S13. The behavior of the bionic electroreceptor is simulated through the finite element method. (a) PDMS film and (b) Disordered doped thin films of nanoparticles and PDMS. (As the object (FEP) approaches the elastic electret.)

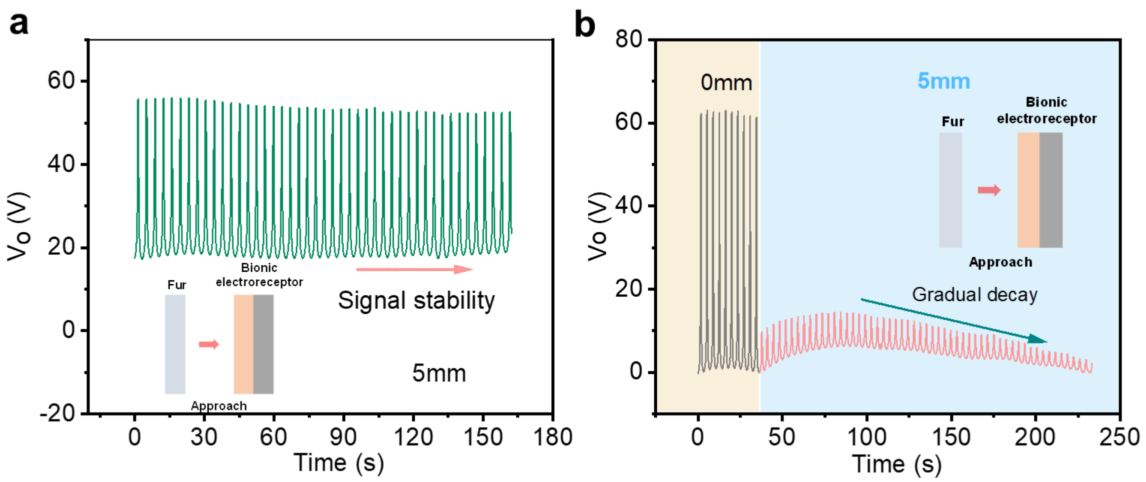

Fig. S14. The output voltage characteristics of the bionic electroreceptor in its non-polarized (a) and polarized (b) states.

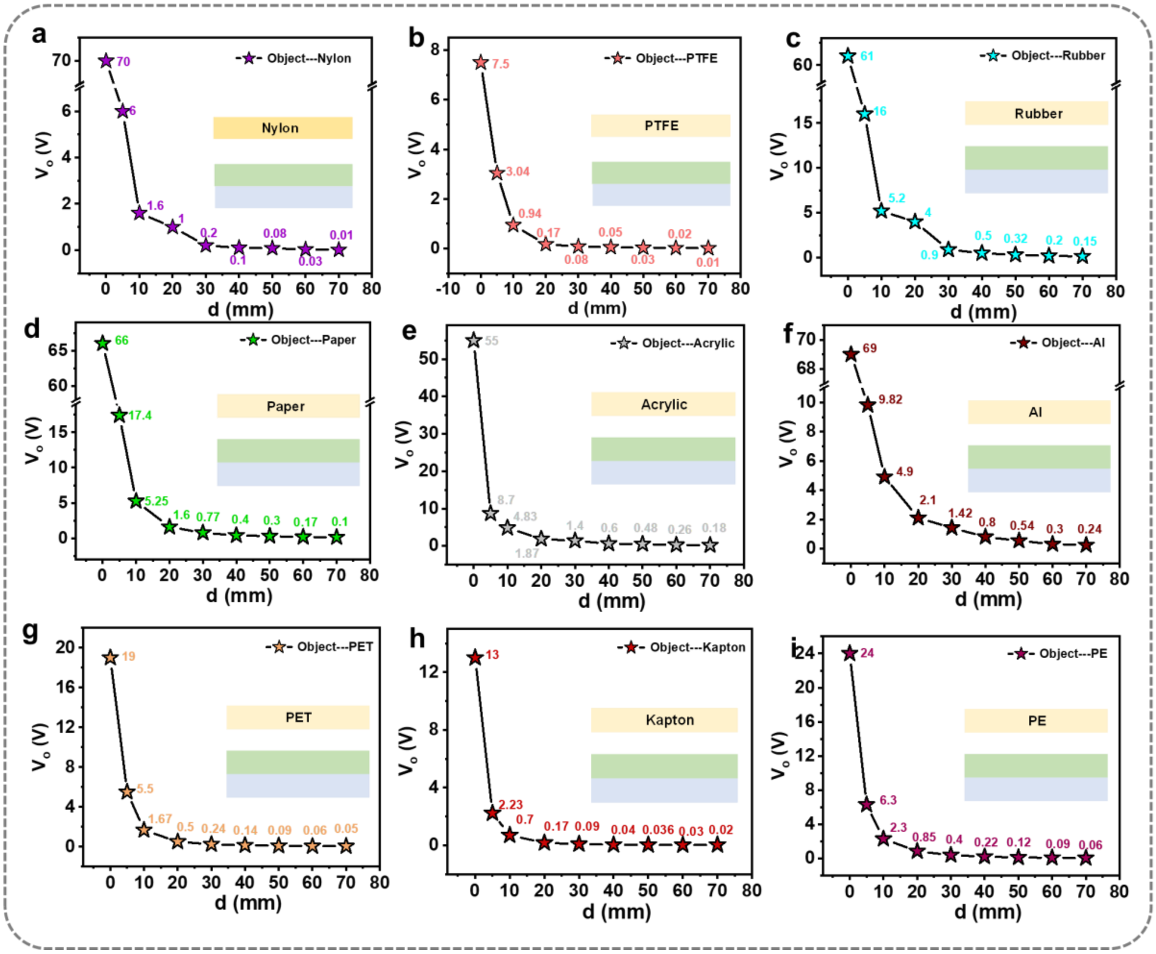

Fig. S15. Different materials were selected as the output voltage of the perception target approaching bionic electroreceptor respectively. (a) Nylon, (b) PTFE, (c) Rubber, (d) Paper, (e) Acrylic, (f) Al, (g) PET, (h) Kapton and (i)PE.

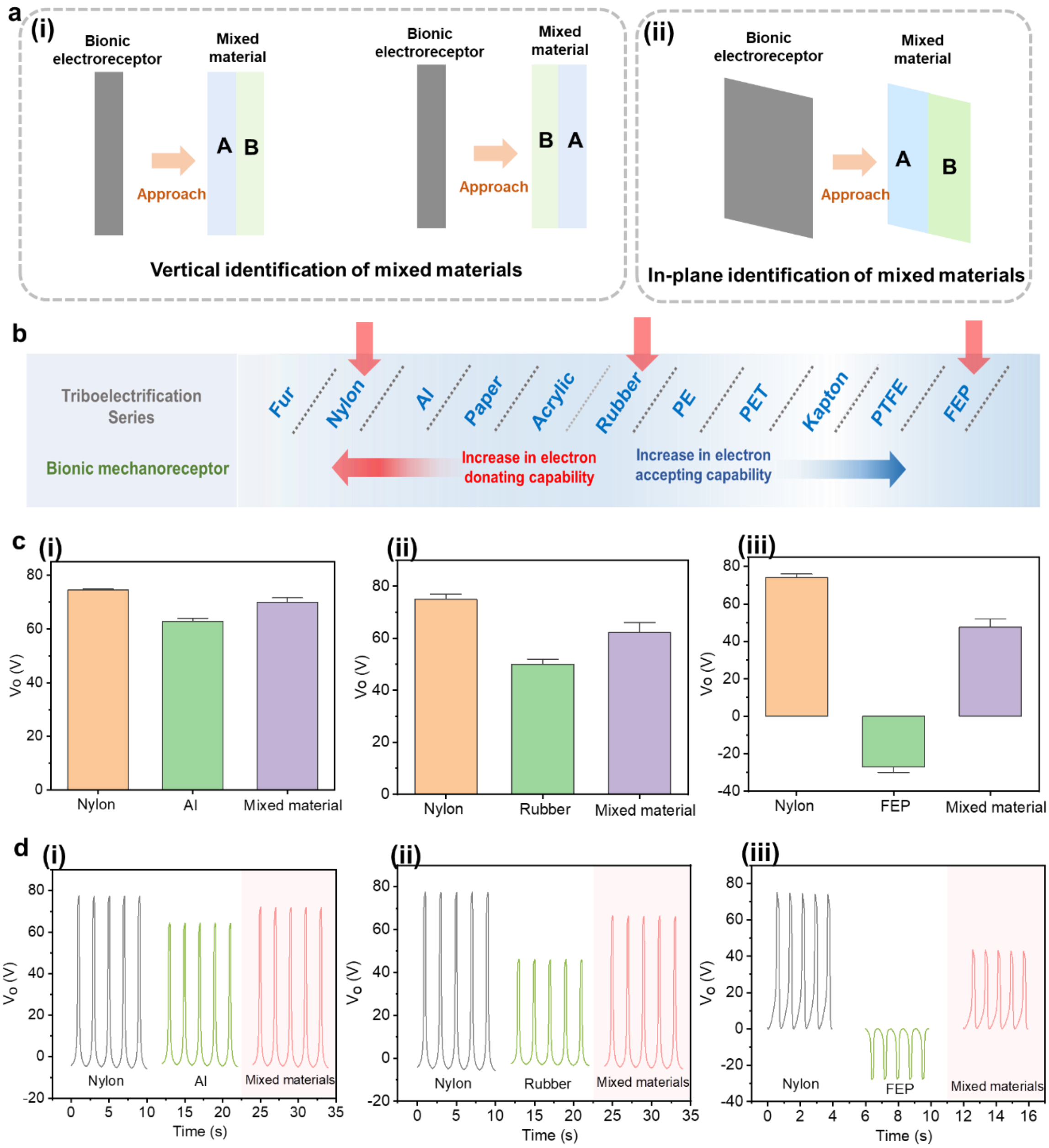

Fig. S16. Identification of mixed materials by the bionic electroreceptor. (a) (i) Schematic of vertical discrimination of mixed materials. (ii) Schematic diagram of the planar differentiation of mixed materials. (b) Selection of mixed materials. (c) Bionic electroreceptor vertically recognizes the output voltage of mixed materials. (d) Bionic electroreceptor vertically recognizes the output voltage of mixed materials.

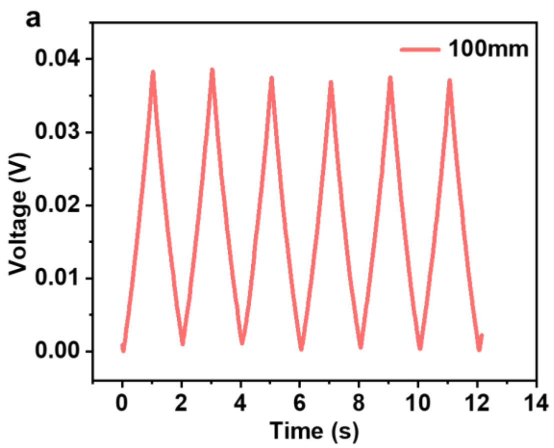

Fig. S17. The output voltage when the bionic electroreceptor tele-perception the fur. (a) 100mm, (b) 150mm.

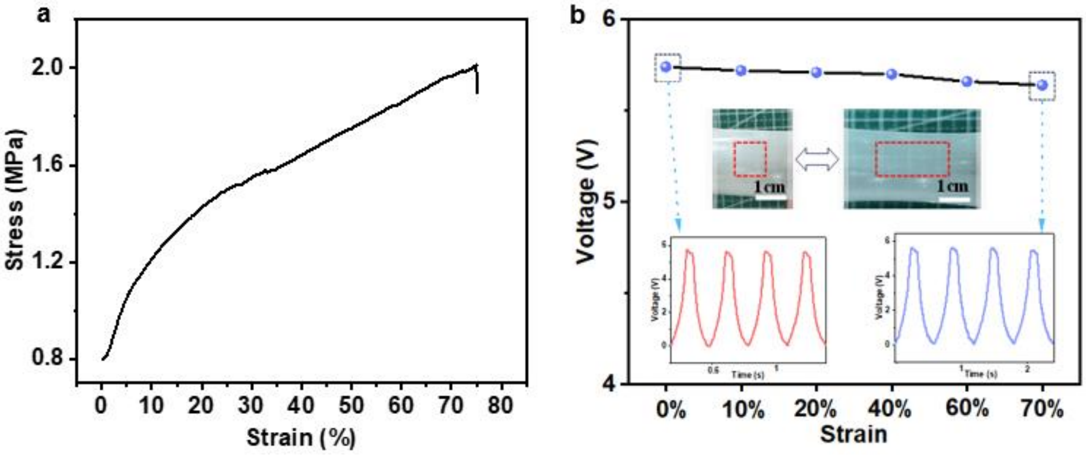

Fig. S18. The elastic properties of the bionic electroreceptor. (a) The strain-stress curves. (b) The output of the bionic electroreceptor under tensile conditions.

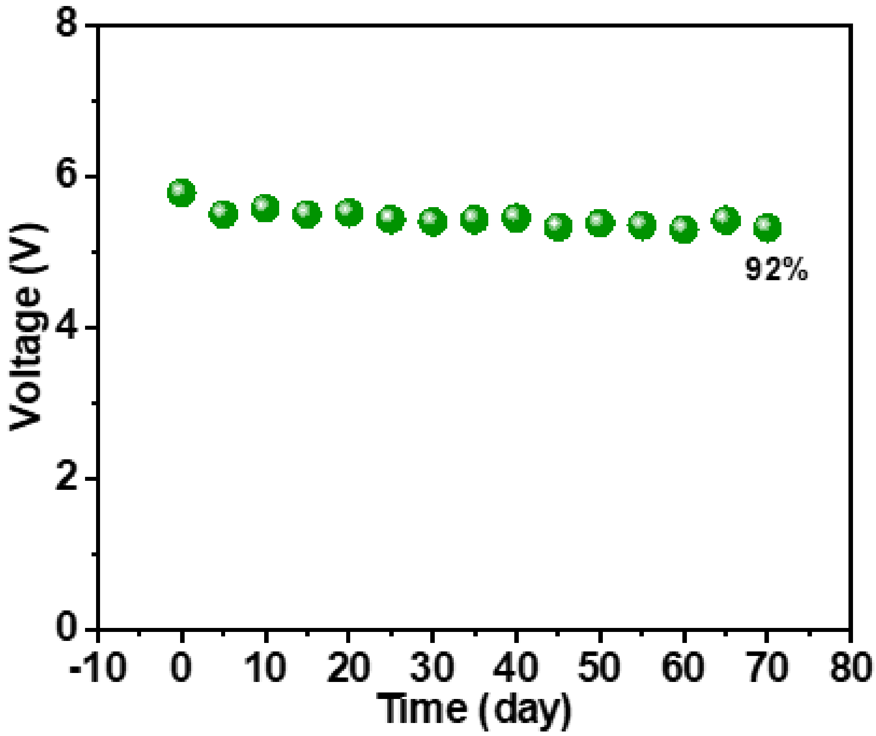

Fig. S19. Durability test the bionic electroreceptor (5mm interval approach).

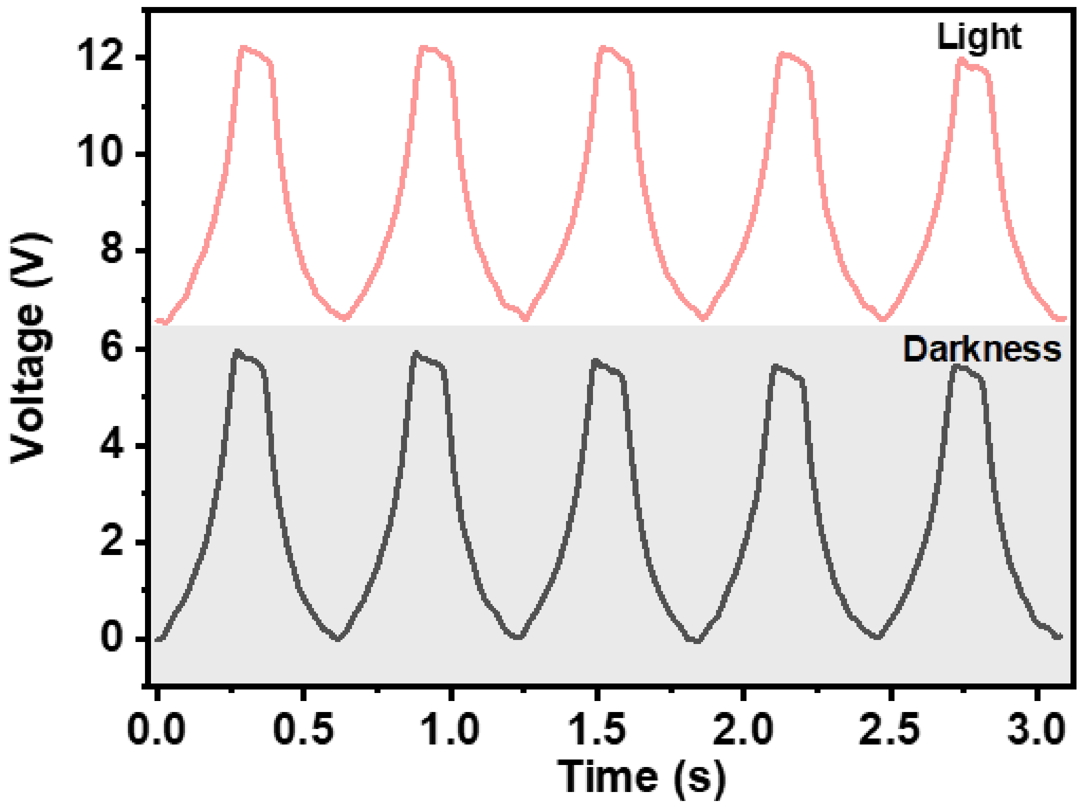

Fig. S20. The output of the bionic electroreceptor under light or darkness conditions.

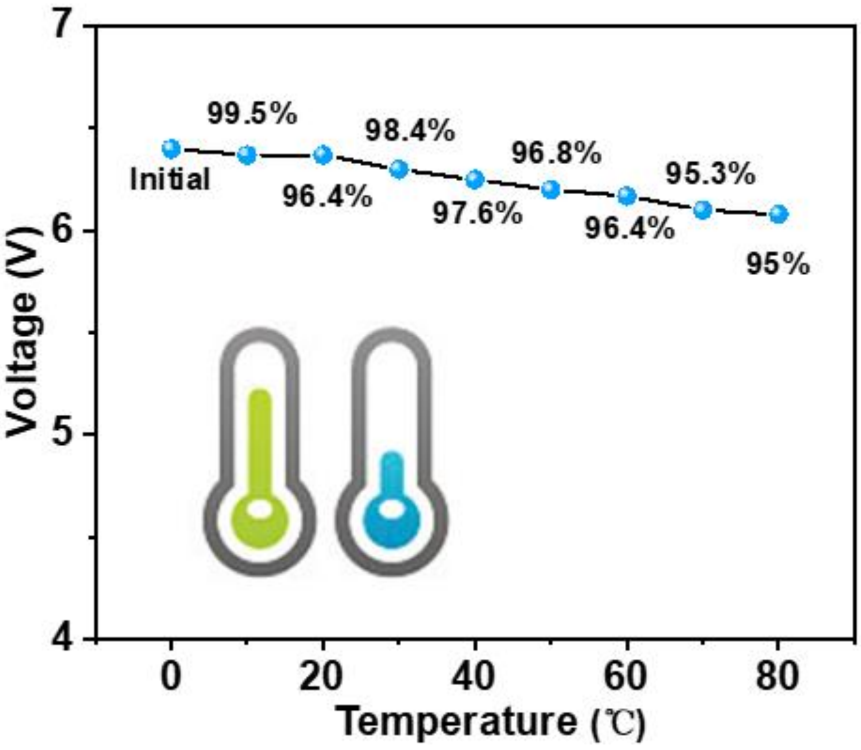

Fig. S21. The output of the bionic electroreceptor at different temperatures.

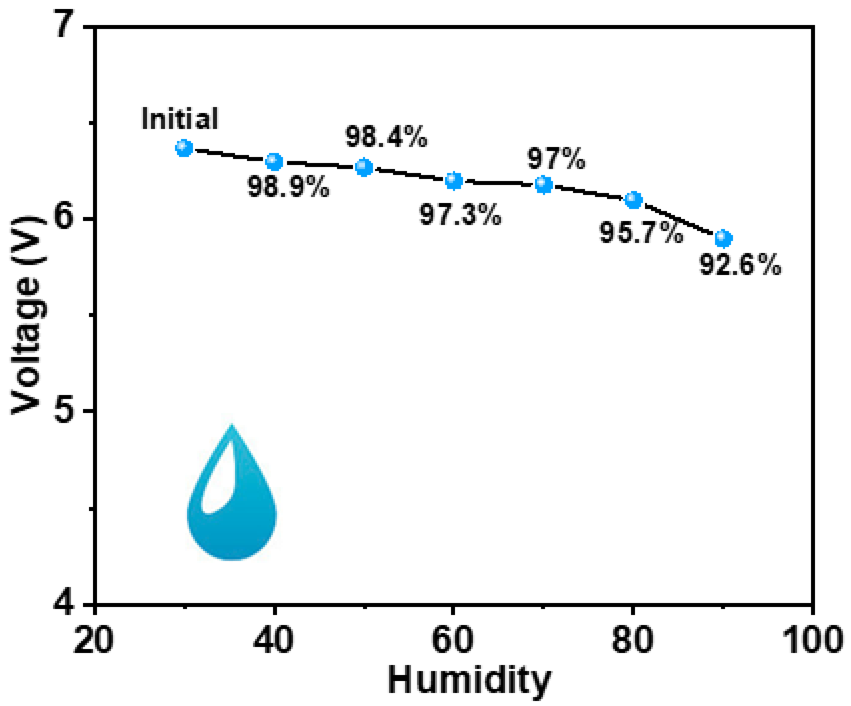

Fig. S22. The output of the bionic electroreceptor at different humidity.

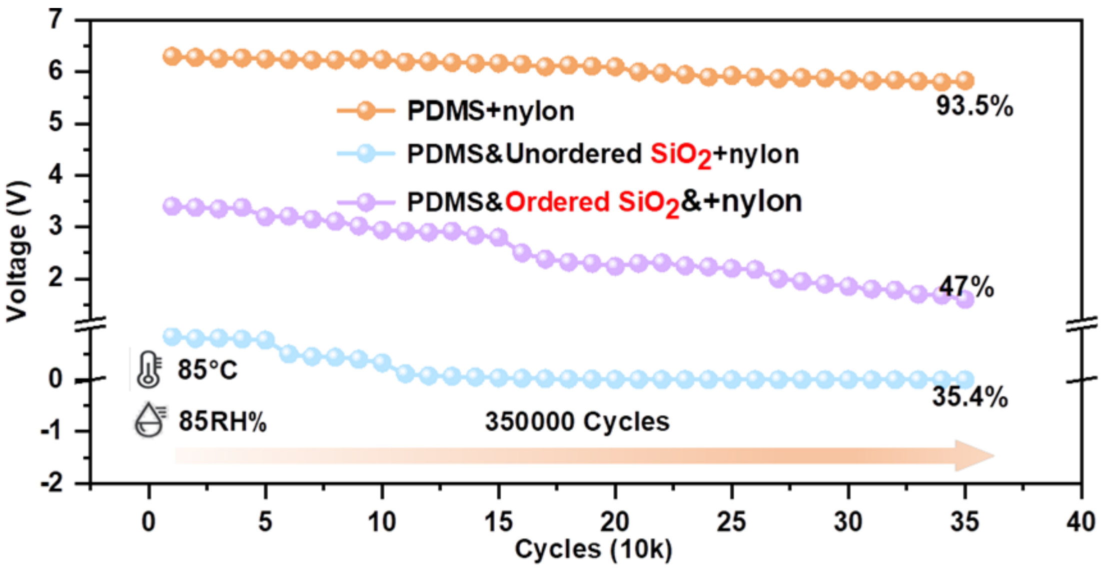

Fig. S23. Experimental comparison of elastomer.

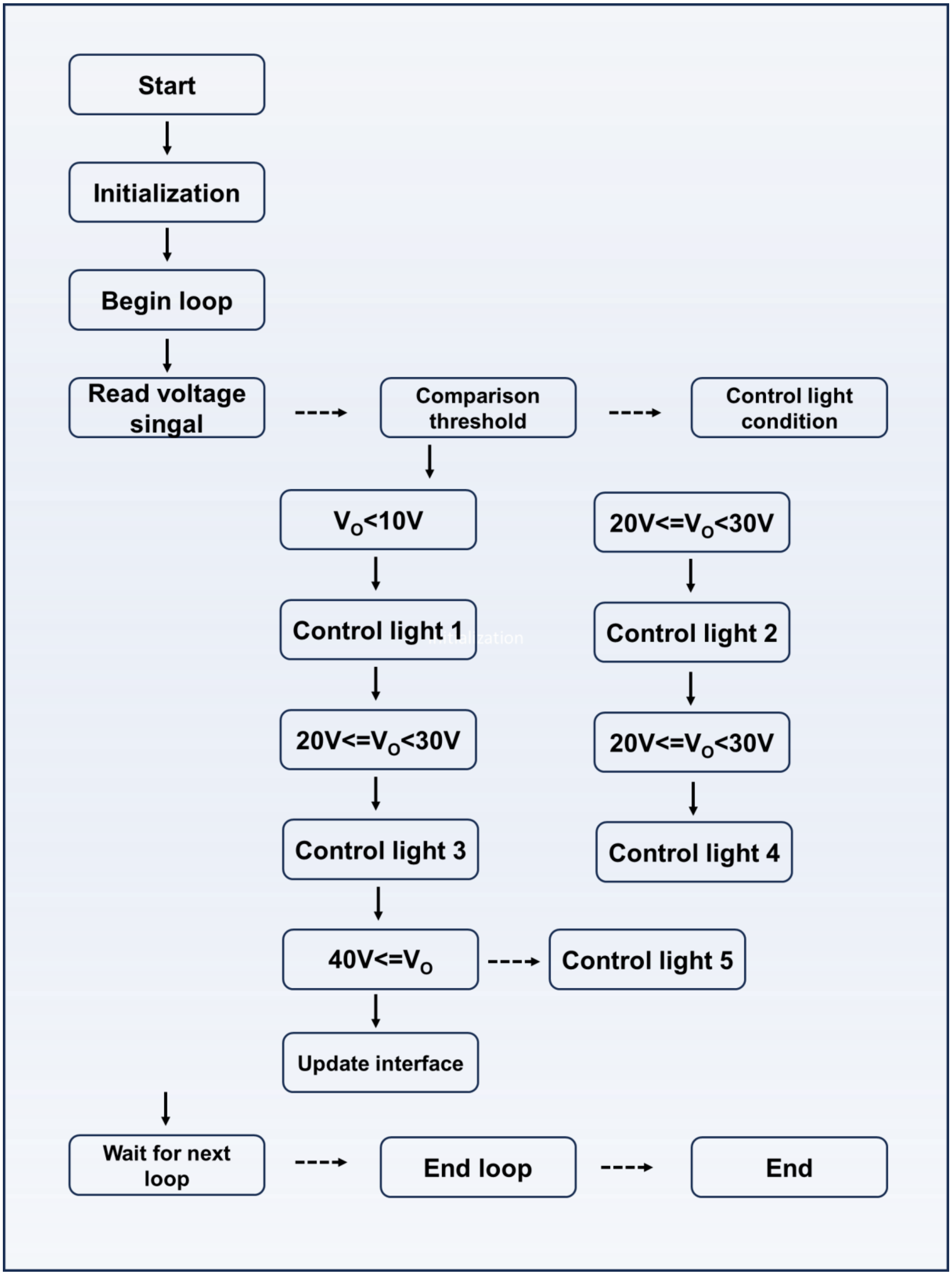

Fig. S24. The workflow of the LabVIEW system.

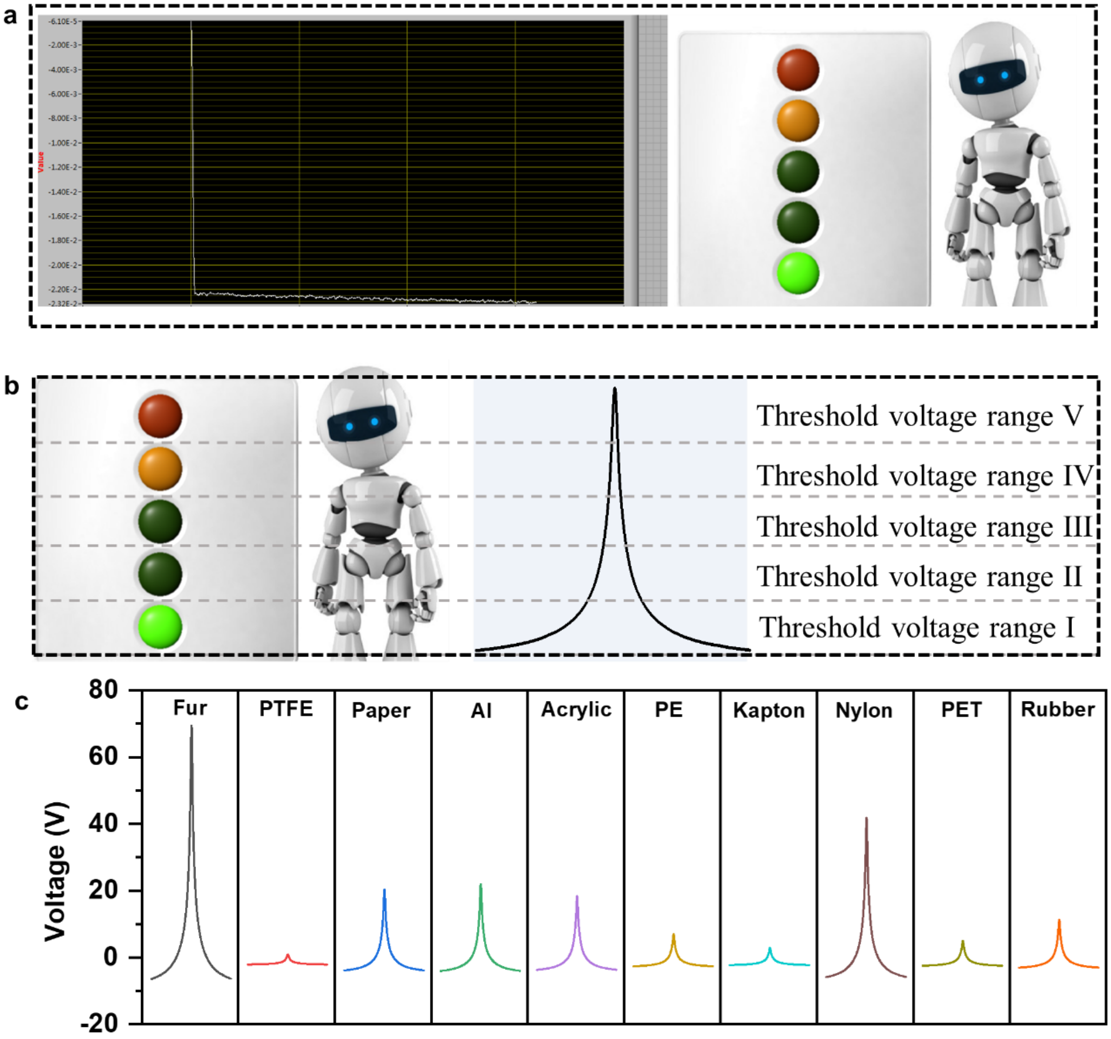

Fig. S25. The selection of the threshold voltage. (a) Virtual robot interface. (b) Setting different threshold voltages will start the virtual robot's lights accordingly. (c) The output of the bionic electroreceptor when different materials approaching.

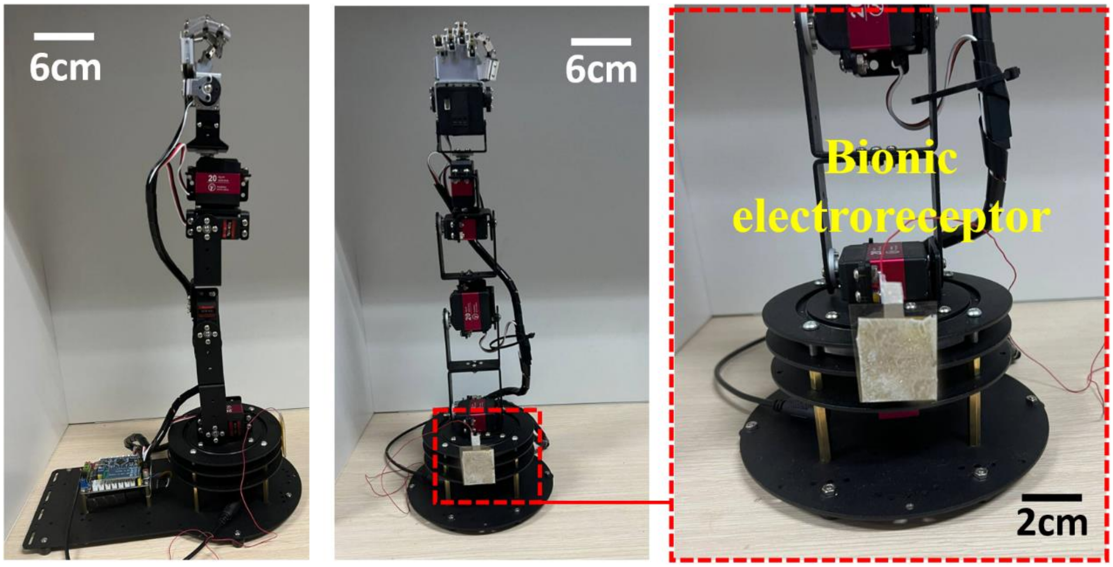

Fig. S26. The photograph of the bionic electroreceptor integrated robot arm.

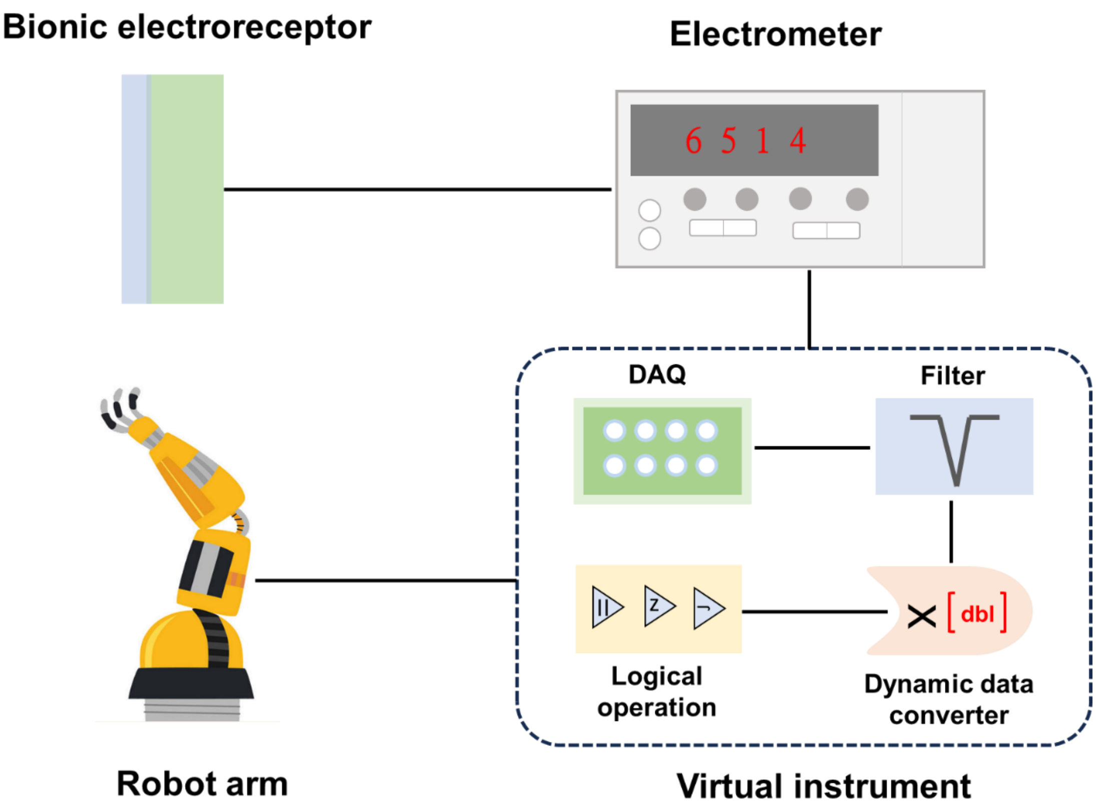

Fig. S27. The structure of the distance perception system. The signals generated by human approaching are detected by the bionic electroreceptor and then transmitted to a virtual instrument which is constructed by a specially written software program (LabVIEW) and consisted of a Data Acquisition (DAQ) module, a filter, a dynamic data converter and logical operation module. The virtual instrument analyzes signals acquired and then delivers appropriate commands to the robot arm.

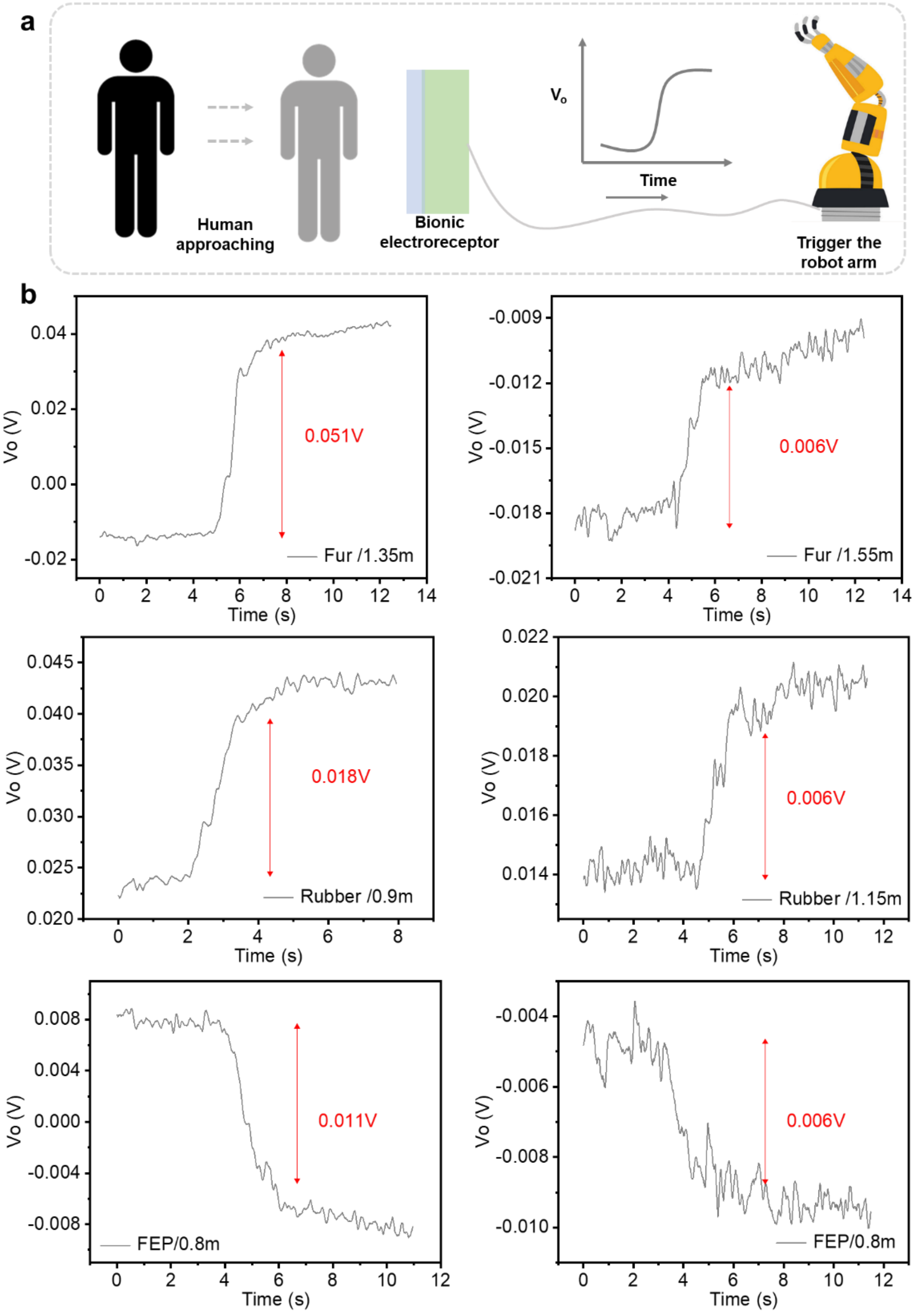

Fig. S28. An example as the human-machine interaction. (a) Human-computer interaction diagram. (b) The voltage signal that accumulated by electrometer when the human approaching the bionic electroreceptor and trigger the robot arm.

a

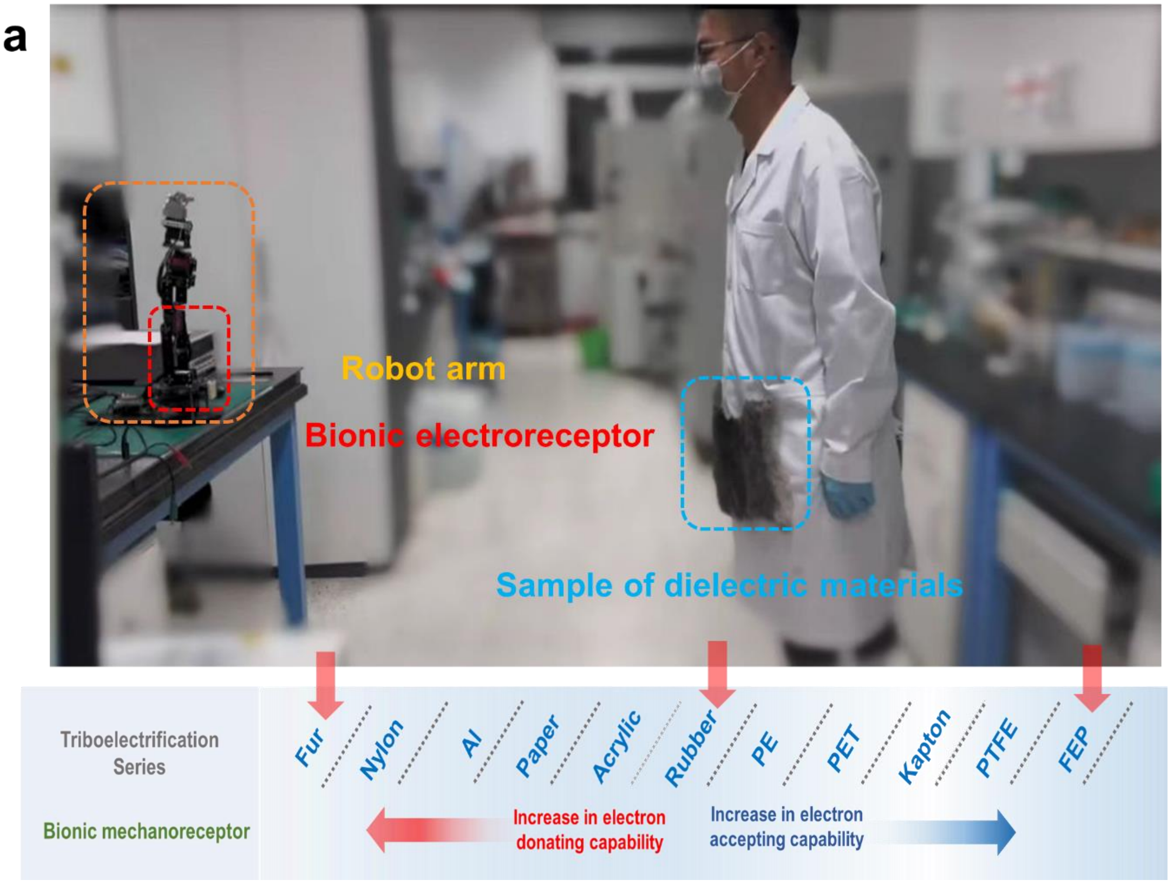
Robot arm
Bionic electroreceptor
Sample of dielectric materials
Triboelectrification Series
Bionic mechanoreceptor
Fur
Nylon
Al
Paper
Acrylic
Rubber
PE
PET
Kapton
PTFE
FEP
Increase in electron donating capability
Increase in electron accepting capability

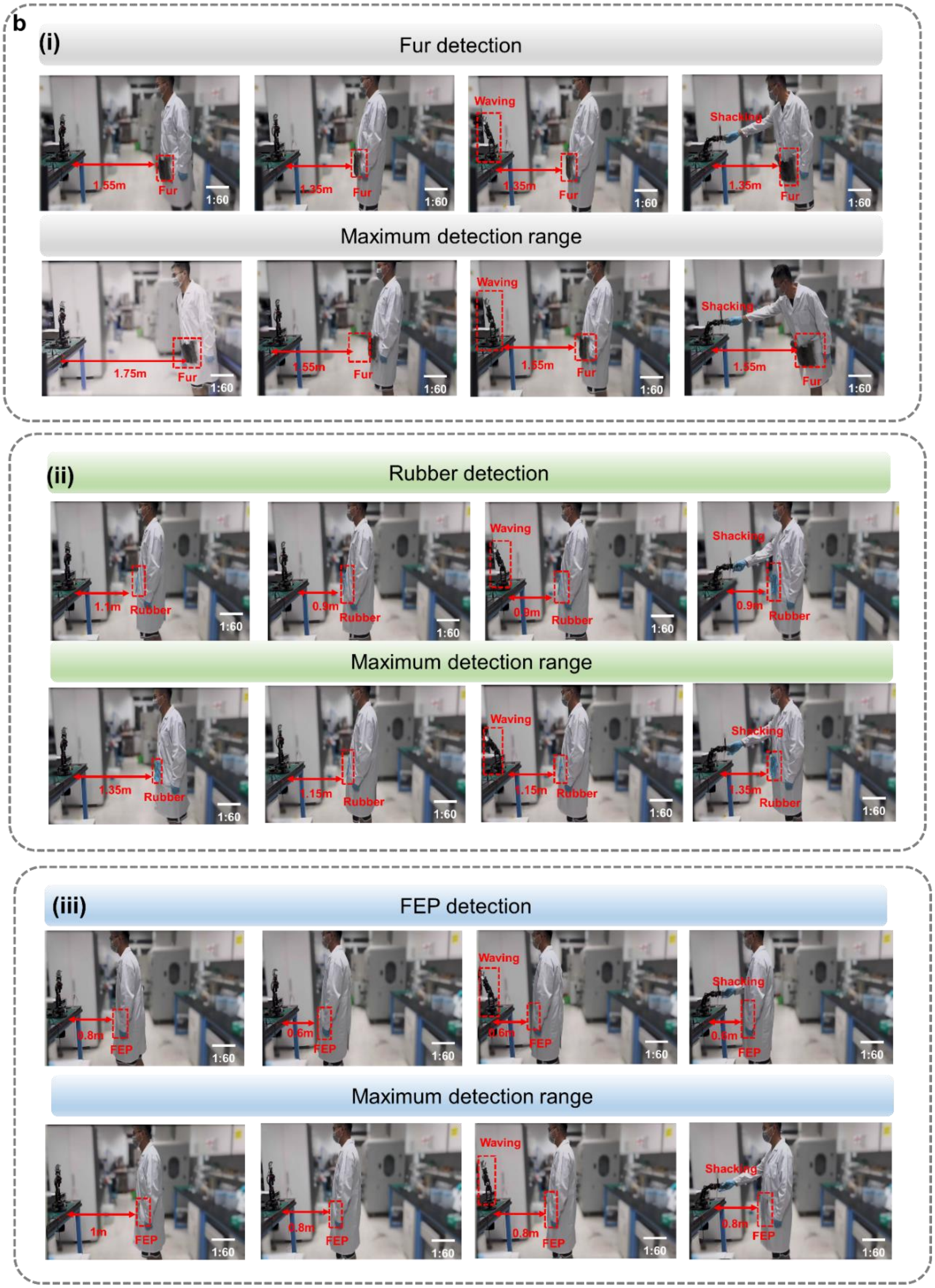

Fig. S29. Tele-perception interaction of wearable materials with a robotic arm. (a) Photograph of tele-perception interaction. (b) Approaching a robotic arm equipped the bionic electroreceptor while wearing (i) Fur (ii) rubber (iii)FEP.

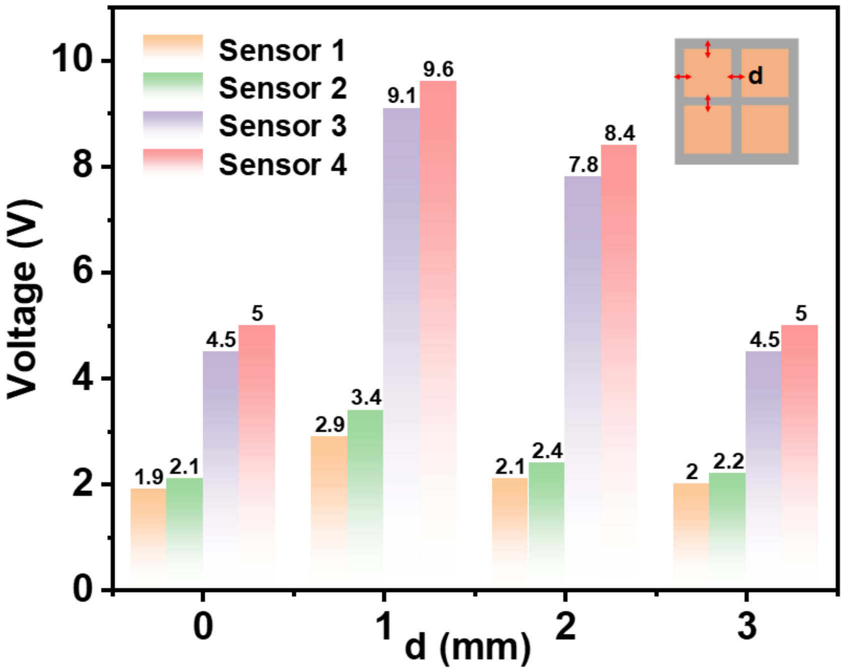

Fig. S30. The separation distance between sensor array effects on voltage values.

```
clc
clear
input_folder = './data/test_data/';
files = dir(fullfile(input_folder, '*.data'));
f = 1;
current_file = fullfile(input_folder, files(f).name);
fid = fopen(current_file, 'r');
C = textscan(fid, repmat('%f', 1, 8), 'Delimiter', ' ', 'HeaderLines', 100)
fclose(fid);
time = C{1}(1:10:end);
voltage = C{2}(1:10:end);

figure
greenColor = [80/255, 127/255, 92/255];
set(groot, 'defaultAxesFontSize', 19);
h0=plot(time, voltage, 'Color', greenColor, 'LineWidth', 2);
hold on;
xlabel('Time(s)', 'FontName', 'Times New Roman');
ylabel('Voltage(V)', 'FontName', 'Times New Roman');
xlim([0 10]);
ylim([-2 2.5]);
set(gca,'FontName', 'Times New Roman');
hold off;
```

Fig. S31. Data visualization code.

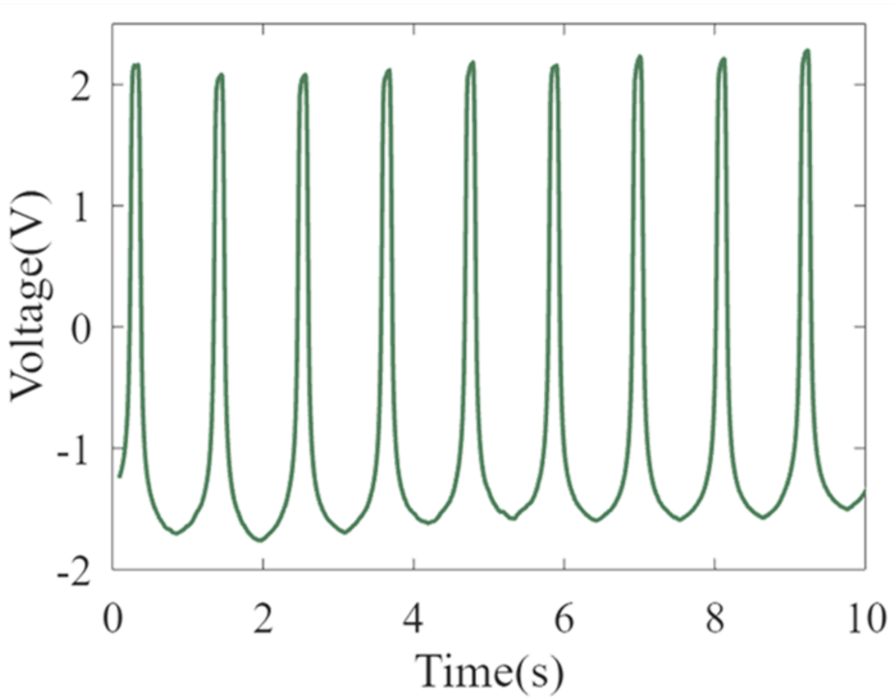

Fig. S32. Data read voltage signal.

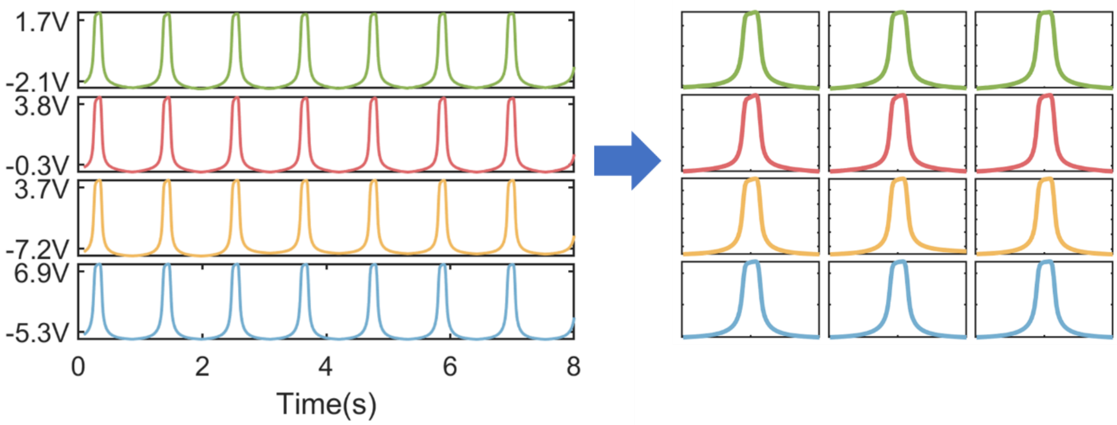

Fig. S33. Raw signal and split signal of acrylic.

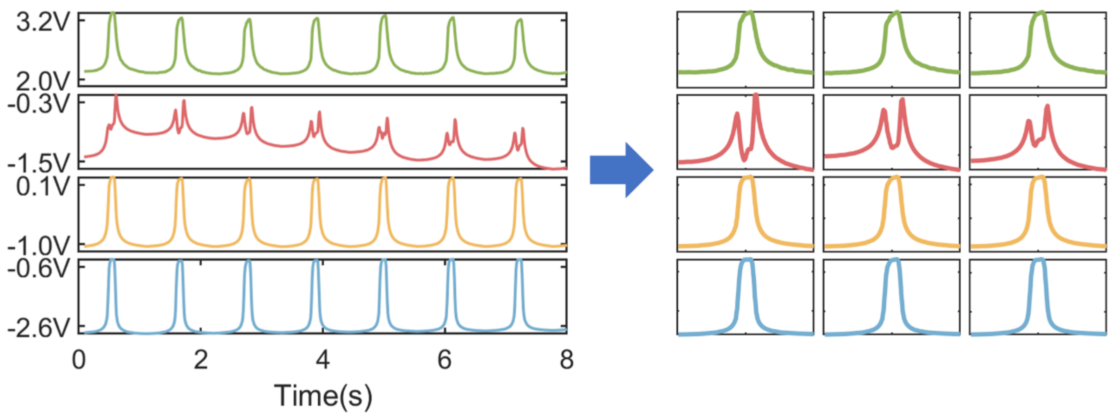

Fig. S34. Raw signal and split signal of Al.

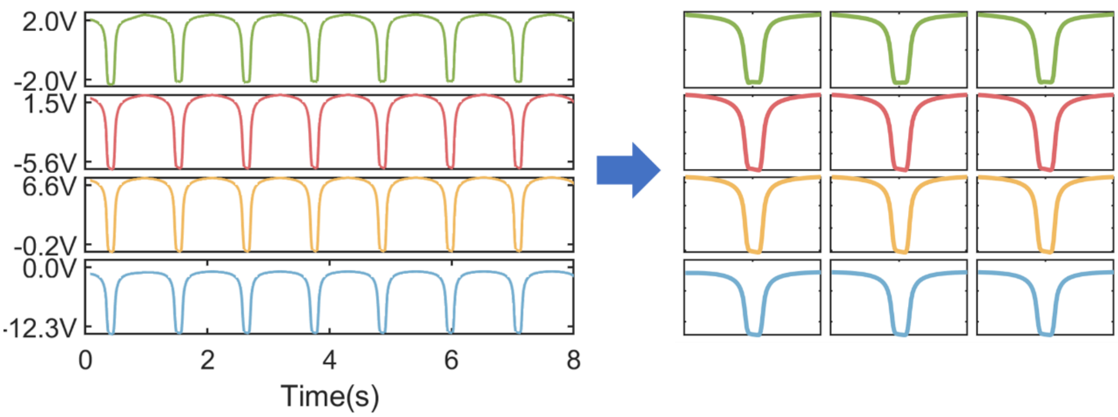

Fig. S35 Raw signal and split signal of FEP.

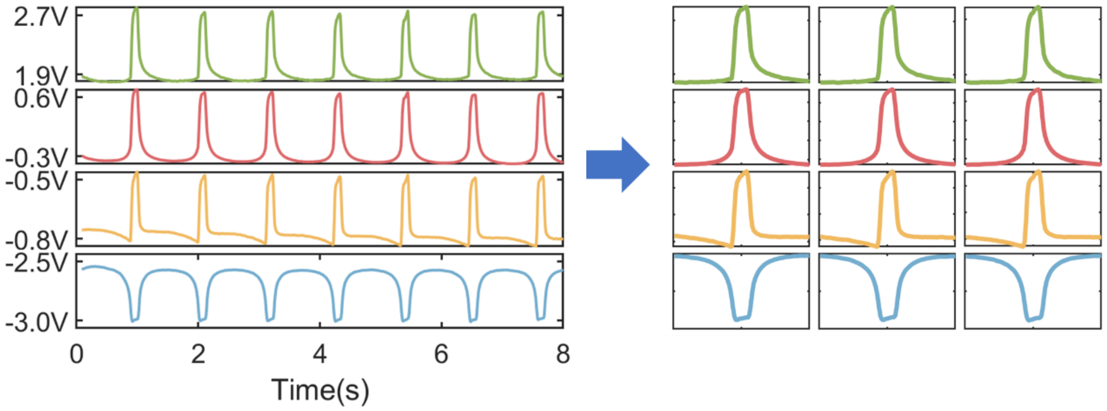

Fig. S36. Raw signal and split signal of kapton.

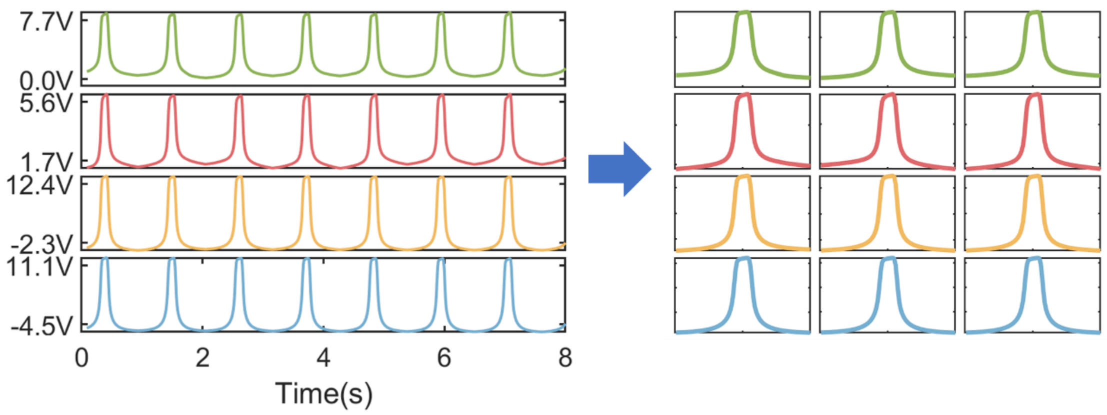

Fig. S37. Raw signal and split signal of nylon.

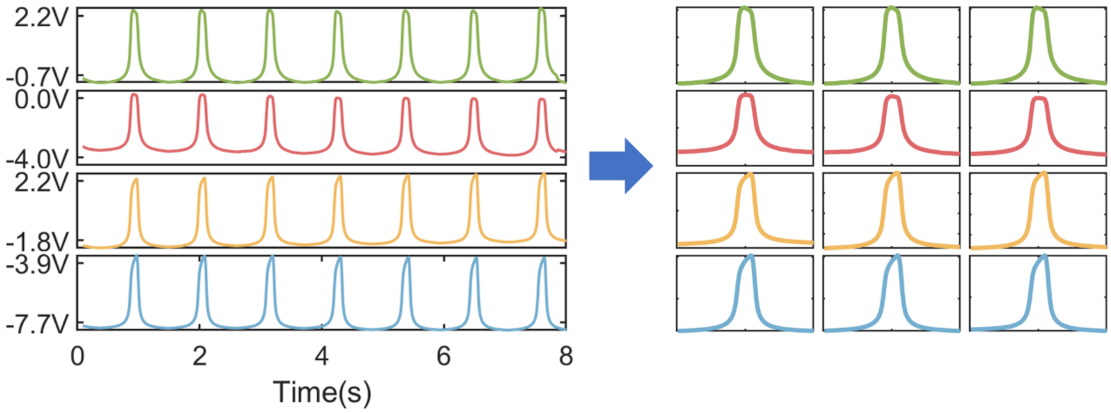

Fig. S38. Raw signal and split signal of paper.

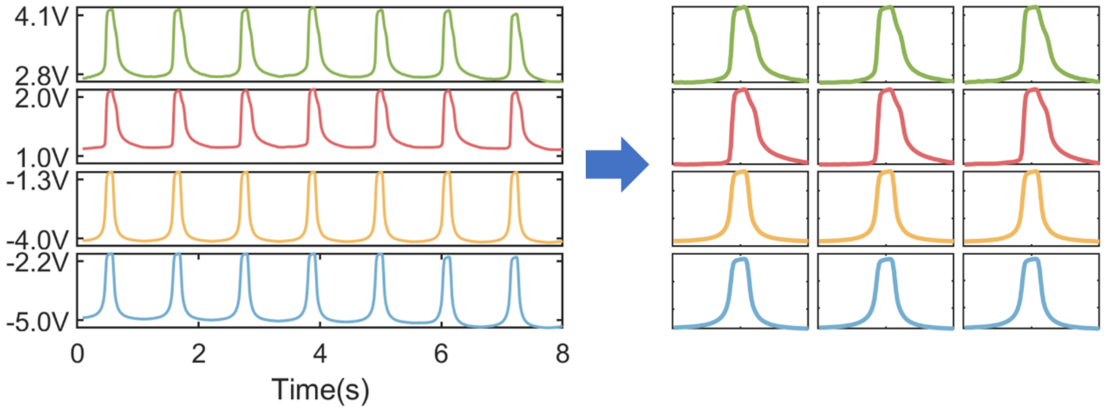

Fig. S39. Raw signal and split signal of PE.

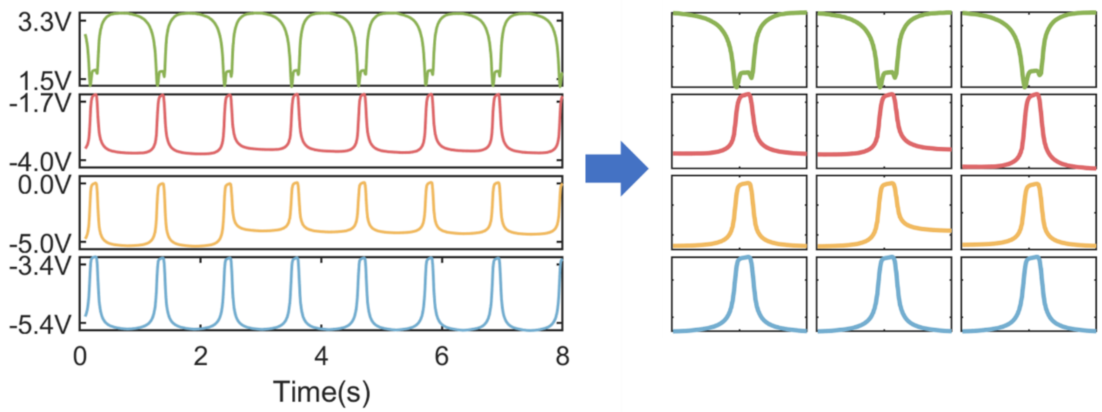

Fig. S40. Raw signal and split signal of PET.

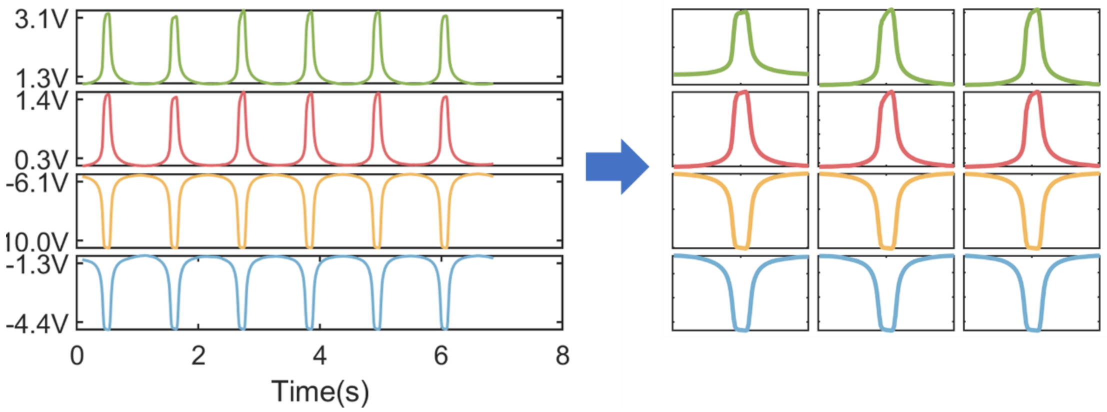

Fig. S41. Raw signal and split signal of PTFE.

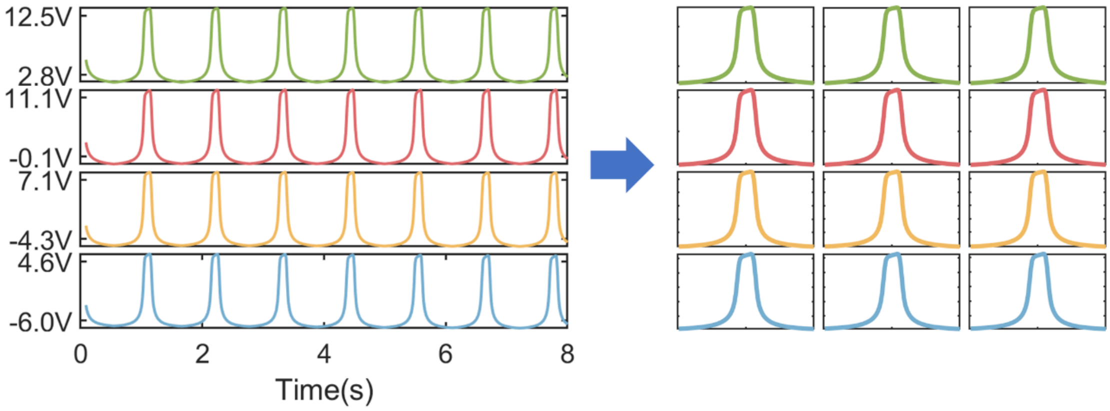

Fig. S42. Raw signal and split signal of rubber.

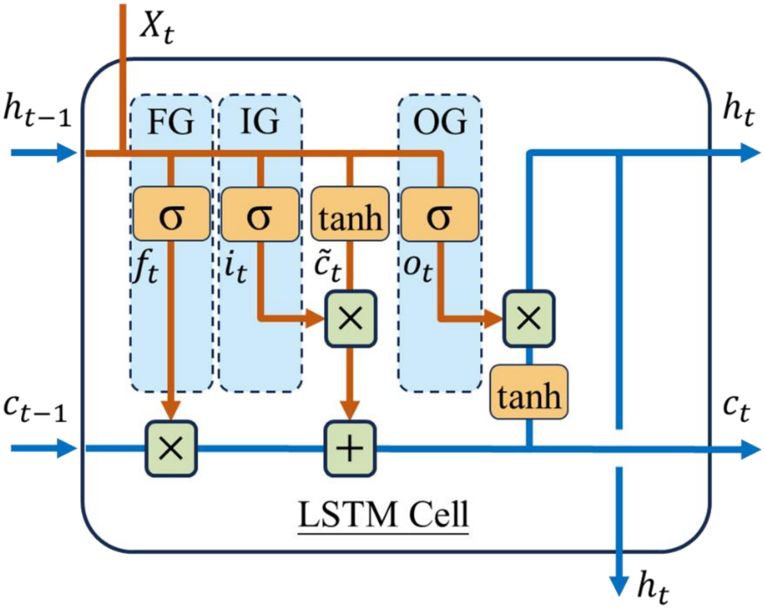

Fig. S43. The structure of the computation unit of Long Short-Term Memory (LSTM) networks.

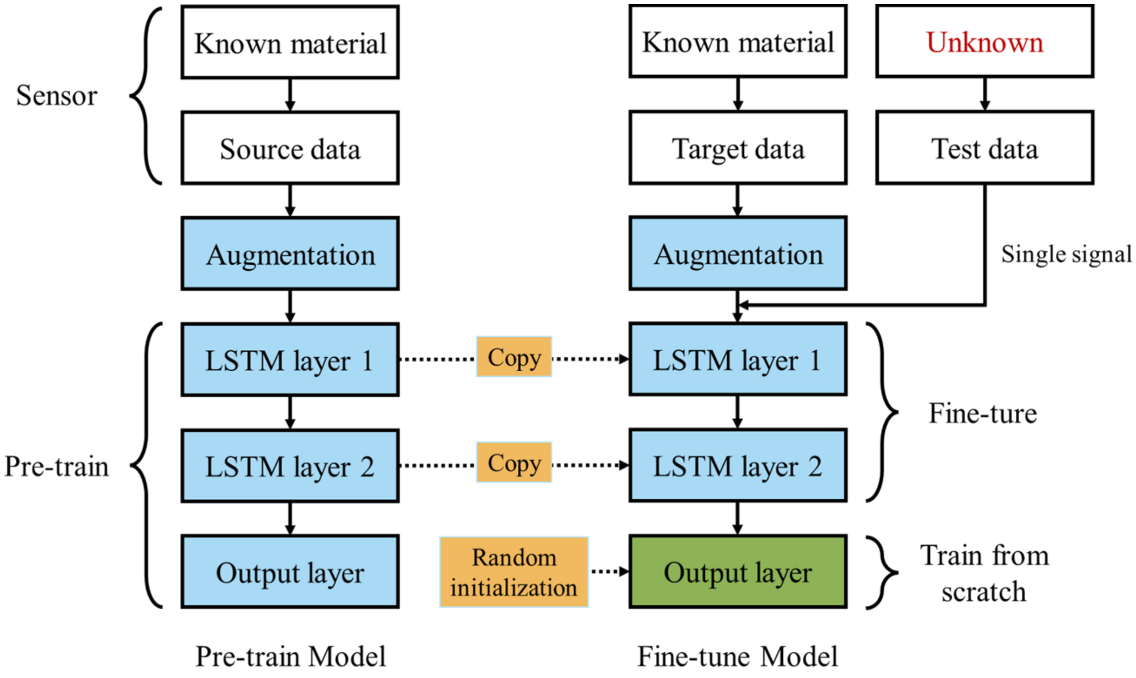

Fig. S44. Fine-tune the training flow.

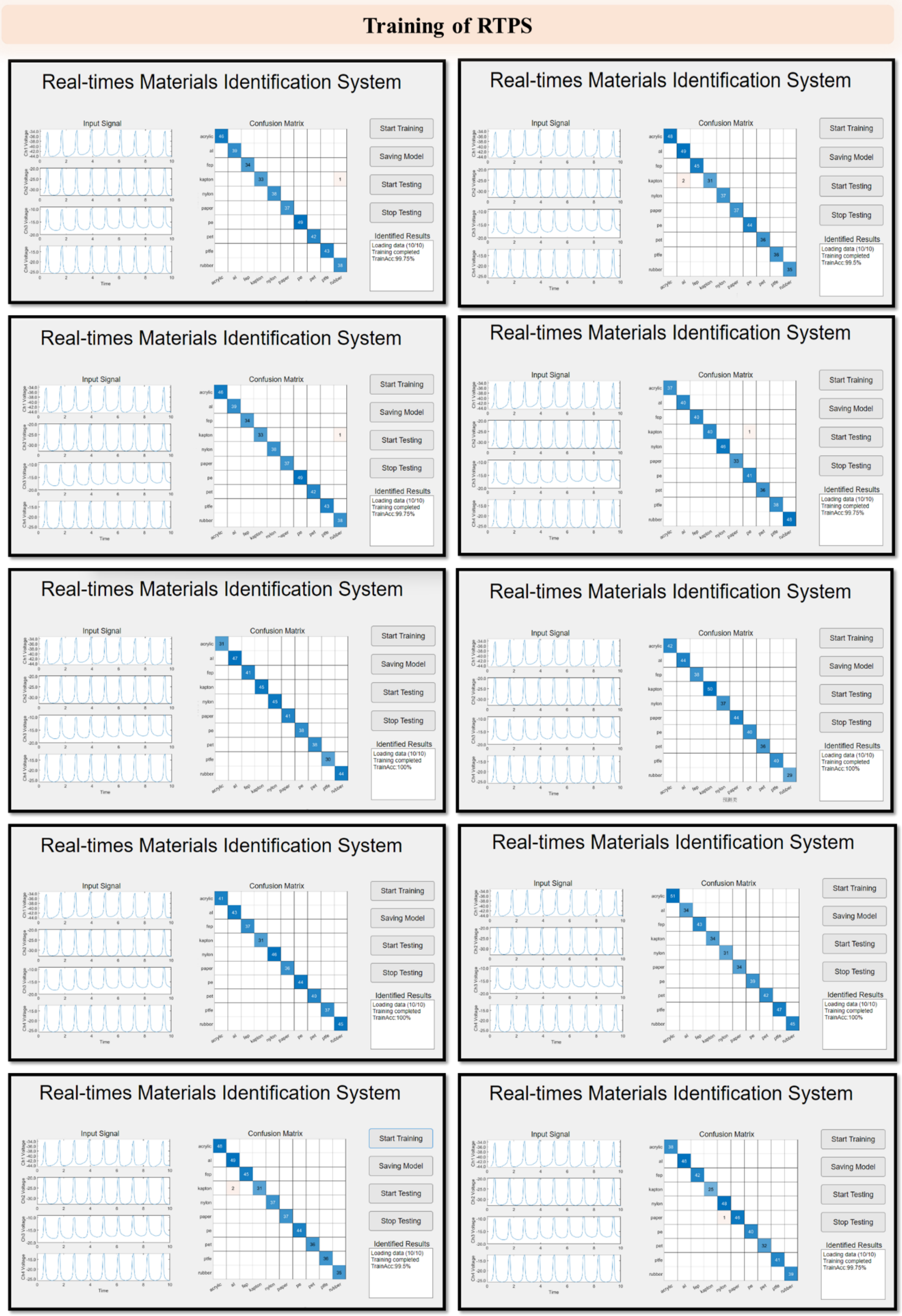

Fig. S45. Training of RTPS.

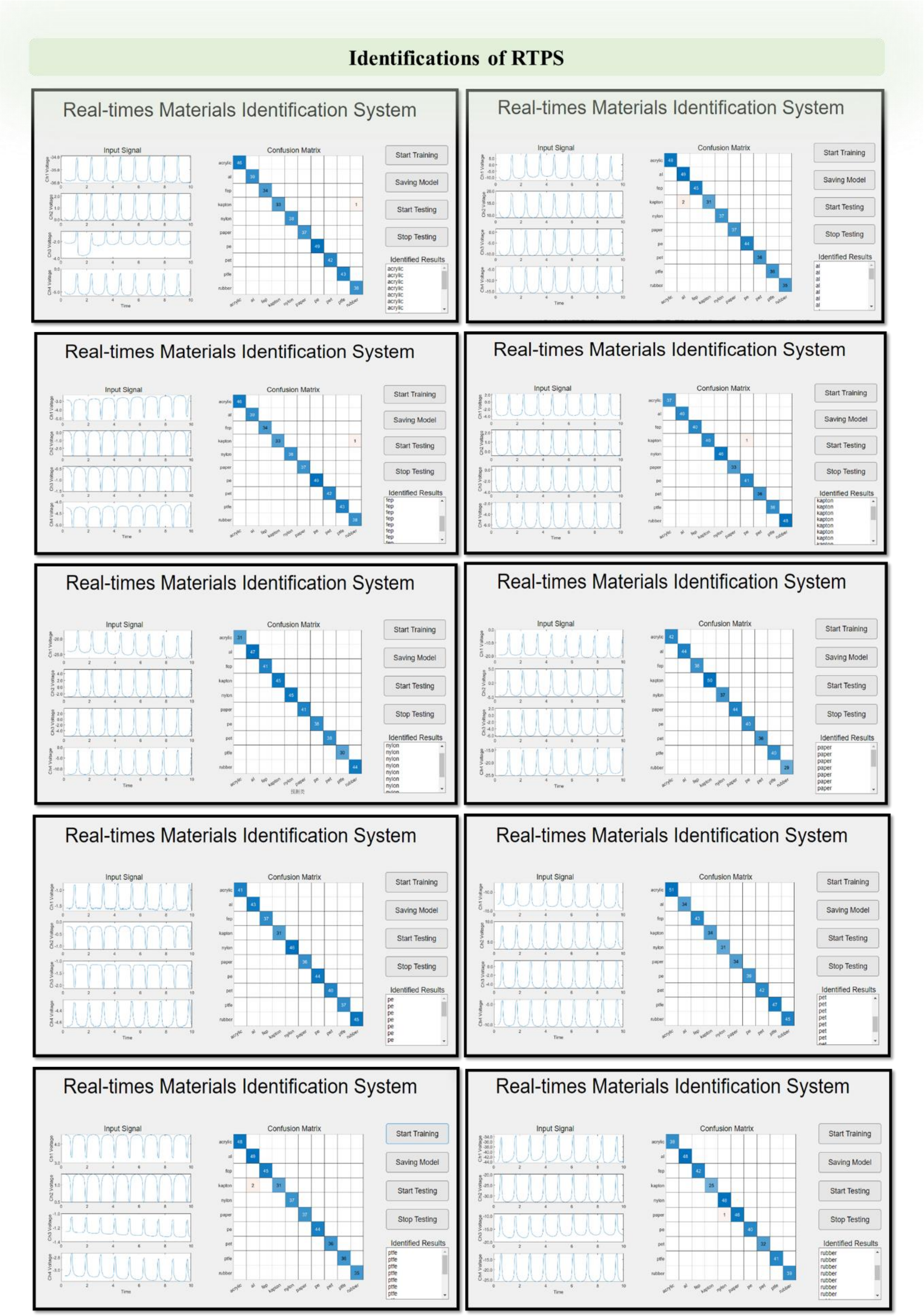

Fig. S46. Identifications of RTPS.

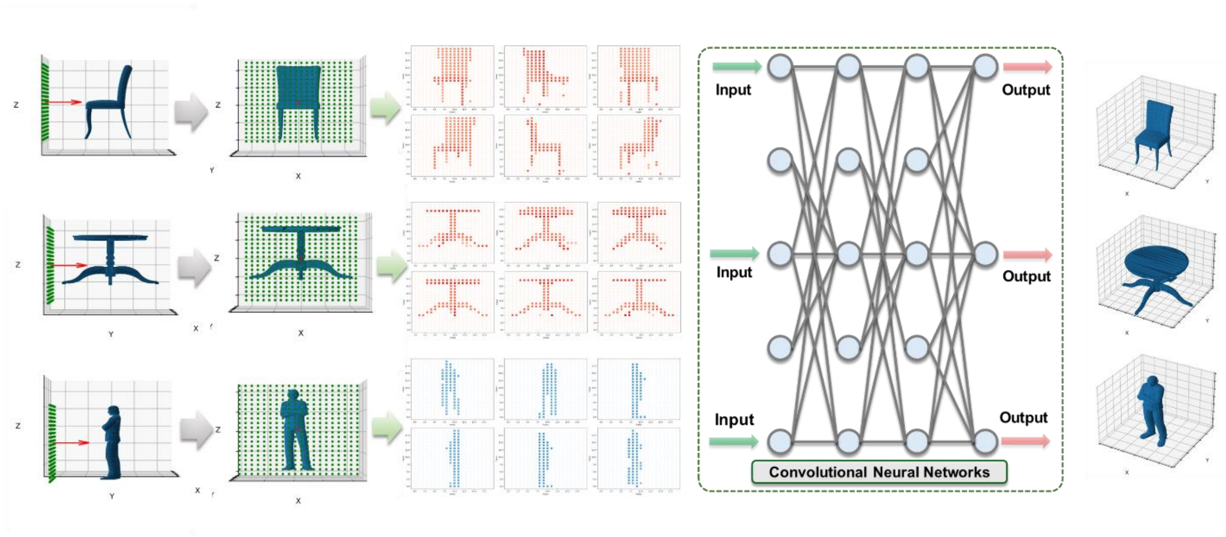

Fig. S47. The 3D object tele-perception system based on the bionic electroreceptor matrix (20 × 20 units) and the CNN (another angle).

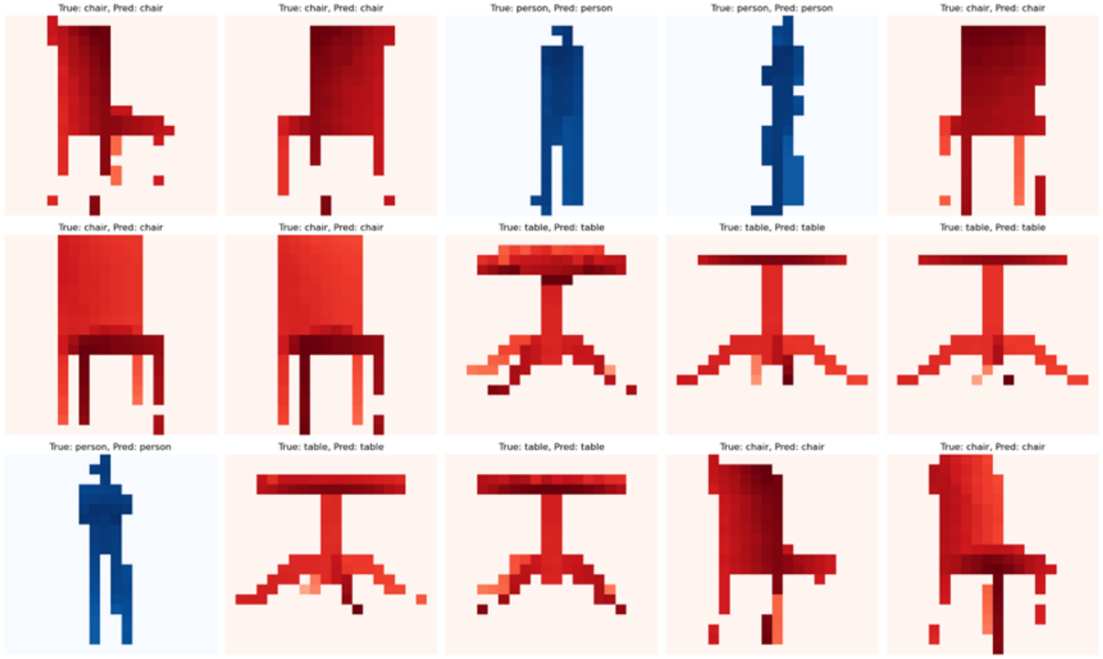

Fig. S48. The recognition performance of the test dataset.

Supplementary Table

Table S1. Comparison of the sensitivity and corresponding device parameters of bionic electroreceptors and their representative works.

	**Dielectric material**	**Electrode material**	**Approaching object**	**ΔV (V)** $_{\approx}$	**Δd (mm)**	**Sensitivity** $_{\approx}$
**1**	Shear stiffening elastomer (SSE)	Conductive shear stiffening gel (c-SSG)	FEP	0.00525	5	0.00105
**2**	-	Cu	PTFE	0.2	50	0.004
**3**	Printing paper	Copper	people	3	200	0.015
**4**	PDMS	Al	people	0.6	10	0.06
**5**	MXene/silicone nanocomposite layer	Conductive sponge	Nylon	10	50	0.2
**6**	PDMS/SiO_2	Organohydrogel	PTFE	8	20	0.35
**7**	-	Cu	FEP	1	2.6	0.38
**8**	PTFE	Cu	Cu	16	40	0.4
**9**	Graphene	ITO	Finger	2.175	5	0.435
**10**	PVDF/$Ti_3C_2T_x$	Cu	PA	10	20	0.5
**11**	Co-NPC/Ecoflex	Mxene/Ecoflex	Human hand	13	20	0.65
**12**	Polyamide&Ag	Cu	Polyamide	20	20	1
**13**	PI-b-C60	Al	Finger	2	1	2
**This work**	PDMS&PTFE/$SrTiO_3$	AgNW	Fur	71	5	14.2

Table S2. Table of parameters for tele-perception of human-computer interaction by a person wearing different materials approaching a robotic arm with bionic electroreceptor.

Material	ΔV (V)	Δd (m)	Sensitivity	Maximum detection range (m)
Fur	0.045	0.2	0.225	1.55
Rubber	0.012	0.25	0.048	1.15
FEP	0.005	0.2	0.0025	0.8

Table S3. Network parameters of the pre-trained model.

Model Structure	Layers	Parameters	Output Shape
Input	Sequence Input	-	(None, Variable)
Block 1	LSTM	Units: 16	(None, 16)
	BN	-	(None, 16)
Block 2	LSTM	Units: 16	(None, 16)
	Dropout	Rate: 0.5	(None, 16)
Output	FC	Units: 10	(None, 10)
	Softmax	-	(None, 10)
	Classification	-	(None, 10)

Supplementary Movies

Movie S1.

The bionic electroreceptor for virtual distance alert robot.

Movie S2.

Interacting with a robot arm through the bionic electroreceptor.

Movie S3.

Real-time materials identification system.

REFERENCES AND NOTES

1. S. Collins, A. Ruina, R. Tedrake, M. Wisse, Efficient Bipedal Robots Based on Passive-Dynamic Walkers. *Science* **307**, 1082-1085 (2005).

2. G. Metta, L. Natale, F. Nori, G. Sandini, D. Vernon, L. Fadiga, C. von Hofsten, K. Rosander, M. Lopes, J. Santos-Victor, A. Bernardino, L. Montesano, The iCub humanoid robot: An open-systems platform for research in cognitive development. *Neural Networks* **23**, 1125-1134 (2010).

3. H. Yousef, M. Boukallel, K. Althoefer, Tactile sensing for dexterous in-hand manipulation in robotics—A review. *Sensors and Actuators A: Physical* **167**, 171-187 (2011).

4. W. Ding, A. C. Wang, C. Wu, H. Guo, Z. L. Wang, Human–Machine Interfacing Enabled by Triboelectric Nanogenerators and Tribotronics. *Advanced Materials Technologies* **4**, 1800487 (2019).

5. J.-K. Song, D. Son, J. Kim, Y. J. Yoo, G. J. Lee, L. Wang, M. K. Choi, J. Yang, M. Lee, K. Do, J. H. Koo, N. Lu, J. H. Kim, T. Hyeon, Y. M. Song, D.-H. Kim, Wearable Force Touch Sensor Array Using a Flexible and Transparent Electrode. *Advanced Functional Materials* **27**, 1605286 (2017).

6. J. Kim, M. Kim, M.-S. Lee, K. Kim, S. Ji, Y.-T. Kim, J. Park, K. Na, K.-H. Bae, H. Kyun Kim, F. Bien, C. Young Lee, J.-U. Park, Wearable smart sensor systems integrated on soft contact lenses for wireless ocular diagnostics. *Nature Communications* **8**, 14997 (2017).

7. S. Sundaram, P. Kellnhofer, Y. Li, J.-Y. Zhu, A. Torralba, W. Matusik, Learning the signatures of the human grasp using a scalable tactile glove. *Nature* **569**, 698-702 (2019).

8. H. Clevers, Modeling Development and Disease with Organoids. *Cell* **165**, 1586-1597 (2016).

9. X. Pu, M. Liu, X. Chen, J. Sun, C. Du, Y. Zhang, J. Zhai, W. Hu, Z. L. Wang, Ultrastretchable, transparent triboelectric nanogenerator as electronic skin for biomechanical energy harvesting and tactile sensing. *Science Advances* **3**, e1700015.

10. T. Jin, Z. Sun, L. Li, Q. Zhang, M. Zhu, Z. Zhang, G. Yuan, T. Chen, Y. Tian, X. Hou, C. Lee, Triboelectric nanogenerator sensors for soft robotics aiming at digital twin applications. *Nature Communications* **11**, 5381 (2020).

11. Y. Yang, H. Zhang, Z.-H. Lin, Y. S. Zhou, Q. Jing, Y. Su, J. Yang, J. Chen, C. Hu, Z. L. Wang, Human Skin Based Triboelectric Nanogenerators for Harvesting Biomechanical Energy and as Self-Powered Active Tactile Sensor System. *ACS Nano* **7**, 9213-9222 (2013).

12. W. Gao, H. Ota, D. Kiriya, K. Takei, A. Javey, Flexible Electronics toward Wearable Sensing. *Accounts of Chemical Research* **52**, 523-533 (2019).

13. D. Kang, P. V. Pikhitsa, Y. W. Choi, C. Lee, S. S. Shin, L. Piao, B. Park, K.-Y. Suh, T.-i. Kim, M. Choi, Ultrasensitive mechanical crack-based sensor inspired by the spider sensory system. *Nature* **516**, 222-226 (2014).

14. T. Sekitani, T. Yokota, U. Zschieschang, H. Klauk, S. Bauer, K. Takeuchi, M. Takamiya, T. Sakurai, T. Someya, Organic Nonvolatile Memory Transistors for Flexible Sensor Arrays. *Science* **326**, 1516-1519 (2009).

15. F. A. Bruno, M. Janneh, A. Gunda, R. Kyselica, P. Stajanca, S. Werzinger, G. Gruca, N. Rijnveld, G. V. Persiano, A. Cutolo, M. Pisco, A. Cusano, Fiber Optic Hydrophones for towed array applications. *Optics and Lasers in Engineering* **160**, 107269 (2023).

16. J. Lan, Y. Xiang, L. Wang, Y. Shi, Vehicle detection and classification by measuring and processing magnetic signal. *Measurement* **44**, 174-180 (2011).

17. T. Wang, Y. Zhou, C. Lei, J. Lei, Z. Yang, Development of an ingenious method for determination of Dynabeads protein A based on a giant magnetoimpedance sensor. *Sensors and Actuators B: Chemical* **186**, 727-733 (2013).

18. H. Fourati, N. Manamanni, L. Afilal, Y. Handrich, Posture and body acceleration tracking by inertial and magnetic sensing: Application in behavioral analysis of free-ranging animals. *Biomedical Signal Processing and Control* **6**, 94-104 (2011).

19. C. Hagleitner, A. Hierlemann, D. Lange, A. Kummer, N. Kerness, O. Brand, H. Baltes, Smart single-chip gas sensor microsystem. *Nature* **414**, 293-296 (2001).

20. S. C. B. Mannsfeld, B. C. K. Tee, R. M. Stoltenberg, C. V. H. H. Chen, S. Barman, B. V. O. Muir, A. N. Sokolov, C. Reese, Z. Bao, Highly sensitive flexible pressure sensors with microstructured rubber dielectric layers. *Nature Materials* **9**, 859-864 (2010).

21. H. Zhang, G. Cao, Z. Wang, Y. Yang, Z. Shi, Z. Gu, Growth of Manganese Oxide Nanoflowers on Vertically-Aligned Carbon Nanotube Arrays for High-Rate Electrochemical Capacitive Energy Storage. *Nano Letters* **8**, 2664-2668 (2008).

22. H. Ren, L. Zheng, G. Wang, X. Gao, Z. Tan, J. Shan, L. Cui, K. Li, M. Jian, L. Zhu, Y. Zhang, H. Peng, D. Wei, Z. Liu, Transfer-Medium-Free Nanofiber-Reinforced Graphene Film and Applications in Wearable Transparent Pressure Sensors. *ACS Nano* **13**, 5541-5548 (2019).

23. W. Wu, L. Wang, Y. Li, F. Zhang, L. Lin, S. Niu, D. Chenet, X. Zhang, Y. Hao, T. F. Heinz, J. Hone, Z. L. Wang, Piezoelectricity of single-atomic-layer MoS2 for energy conversion and piezotronics. *Nature* **514**, 470-474 (2014).

24. X. Y. Kong, Z. L. Wang, Spontaneous Polarization-Induced Nanohelixes, Nanosprings, and Nanorings of Piezoelectric Nanobelts. *Nano Letters* **3**, 1625-1631 (2003).

25. B. D. Argall, A. G. Billard, A survey of Tactile Humanâ€“Robot Interactions. *Robotics and Autonomous Systems* **58**, 1159-1176 (2010).

26. A. Zacharaki, I. Kostavelis, A. Gasteratos, I. Dokas, Safety bounds in human robot interaction: A survey. *Safety Science* **127**, 104667 (2020).

27. M. Salauddin, S. M. S. Rana, M. Sharifuzzaman, S. H. Lee, M. A. Zahed, Y. Do Shin, S. Seonu, H. S. Song, T. Bhatta, J. Y. Park, Laser-carbonized MXene/ZiF-67 nanocomposite as an intermediate layer for boosting the output performance of fabric-based triboelectric nanogenerator. *Nano Energy* **100**, 107462 (2022).

28. G. H. Han, S. H. Lee, J. Gao, H. S. Shin, J. W. Lee, K. J. Choi, Y. Yang, H.-C. Song, Y. Kim, J. M. Baik, Sustainable charged composites with amphiphobic surfaces for harsh environmentâ€"tolerant non-contact mode triboelectric nanogenerators. *Nano Energy* **112**, 108428 (2023).

29. S. Yu, H. Zhang, J. Zhang, Z. Hu, High-sensitivity RGO-TiO2 humidity sensor driven by triboelectric nanogenerators for non-contact monitoring of human respiration. *Journal of Alloys and Compounds* **935**, 168006 (2023).

30. A. Nawaz, M. Kang, H. W. Choi, R. T. M. Ahmad, S.-W. Kim, D. H. Yoon, ZnFe2O4 nanocomposite films for electromagnetic-triboelectric-piezoelectric effect-based hybrid multimodal nanogenerator. *Chemical Engineering Journal* **454**, 140262 (2023).

31. Z. Zhou, W. Yuan, Functionally integrated conductive organohydrogel sensor for wearable motion detection, triboelectric nanogenerator and non-contact sensing. *Composites Part A: Applied Science and Manufacturing* **172**, 107603 (2023).

32. J. W. Lee, S. Jung, J. Jo, G. H. Han, D.-M. Lee, J. Oh, H. J. Hwang, D. Choi, S.-W. Kim, J. H. Lee, C. Yang, J. M. Baik, Sustainable highly charged C60-functionalized polyimide in a non-contact mode triboelectric nanogenerator. *Energy Environ. Sci.* **14**, 1004-1015 (2021).

33. H. L. Wang, L. Su, H. Y. Li, Z. L. Wang, G. Zhu, Electret-induced electric field assisted luminescence modulation for interactive visualized sensing in a non-contact mode. *Materials Horizons* **7**, 1144-1149 (2020).

34. X. Li, S. Li, X. Guo, J. Shao, Z. L. Wang, D. Wei, Triboiontronics for efficient energy and information flow. *Matter* **6**, 3912-3926 (2023).

35. Y. Du, S. Fu, C. Shan, H. Wu, W. He, J. Wang, H. Guo, G. Li, Z. Wang, C. Hu, A Novel Design Based on Mechanical Time‐Delay Switch and Charge Space Accumulation for High Output Performance Direct‐Current Triboelectric Nanogenerator. *Advanced Functional Materials* **32**, (2022).

36. Z. H. Guo, H. L. Wang, J. Shao, Y. Shao, L. Jia, L. Li, X. Pu, Z. L. Wang, Bioinspired soft electroreceptors for artificial precontact somatosensation. *Science Advances* **8**, eabo5201.

37. T. Kattenborn, J. Leitloff, F. Schiefer, S. Hinz, Review on Convolutional Neural Networks (CNN) in vegetation remote sensing. *ISPRS Journal of Photogrammetry and Remote Sensing* **173**, 24-49 (2021).

38. A. Holzinger, B. Malle, A. Saranti, B. Pfeifer, Towards multi-modal causability with Graph Neural Networks enabling information fusion for explainable AI. *Information Fusion* **71**, 28-37 (2021).

39. S. H. Kim, G. W. Baek, J. Yoon, S. Seo, J. Park, D. Hahm, J. H. Chang, D. Seong, H. Seo, S. Oh, K. Kim, H. Jung, Y. Oh, H. W. Baac, B. Alimkhanuly, W. K. Bae, S. Lee, M. Lee, J. Kwak, J. H. Park, D. Son, A Bioinspired Stretchable Sensory-Neuromorphic System. *Adv. Mater.* **33**, e2104690 (2021).

40. J. K. Song, J. Kim, J. Yoon, J. H. Koo, H. Jung, K. Kang, S. H. Sunwoo, S. Yoo, H. Chang, J. Jo, W. Baek, S. Lee, M. Lee, H. J. Kim, M. Shin, Y. J. Yoo, Y. M. Song, T. Hyeon, D. H. Kim, D. Son, Stretchable colour-sensitive quantum dot nanocomposites for shape-tunable multiplexed phototransistor arrays. *Nat Nanotechnol* **17**, 849-856 (2022).

41. X. T. Zheng, Z. Yang, L. Sutarlie, M. Thangaveloo, Y. Yu, N. A. B. M. Salleh, J. S. Chin, Z. Xiong, D. L. Becker, X. J. Loh, B. C. K. Tee, X. Su, Battery-free and AI-enabled multiplexed sensor patches for wound monitoring. *Science Advances* **9**, eadg6670.

42. F. Liu, S. Deswal, A. Christou, Y. Sandamirskaya, M. Kaboli, R. Dahiya, Neuro-inspired electronic skin for robots. *Science Robotics* **7**, eabl7344.

43. S. K. Mishra, S. M. Tisel, P. Orestes, S. K. Bhangoo, M. A. Hoon, TRPV1â€• lineage neurons are required for thermal sensation. *The EMBO Journal* **30**, 582-593-593 (2011).

44. T. T. Fjallbrant, P. R. Manger, J. D. Pettigrew, Some related aspects of platypus electroreception: temporal integration behaviour, electroreceptive thresholds and directionality of the bill acting as an antenna. *Philos Trans R Soc Lond B Biol Sci* **353**, 1211-1219 (1998).

45. J. D. Pettigrew, P. R. Manger, S. L. Fine, The sensory world of the platypus. *Philos Trans R Soc Lond B Biol Sci* **353**, 1199-1210 (1998).

46. U. Proske, J. E. Gregory, A. Iggo, Sensory receptors in monotremes. *Philos Trans R Soc Lond B Biol Sci* **353**, 1187-1198 (1998).

47. H. Wang, H. Wang, F. Yang, J. Zhang, Q. Li, M. Zhou, Y. Jiang, Deposition and characterization of large-scale FePt nanoparticle monolayers on SiO2/Si surface. *Surf. Coat. Technol.* **204**, 1509-1513 (2010).

48. E. R. Leite, E. J. H. Lee, C. Ribeiro, E. Longo, Controlled Thickness Deposition of Ultrathin Ceramic Films by Spin Coating. *J. Am. Ceram. Soc.* **89**, 2016-2020 (2006).

49. J. E. Gregory, A. Iggo, A. K. McIntyre, U. Proske, Responses of electroreceptors in the platypus bill to steady and alternating potentials. *The Journal of Physiology* **408**, 391-404 (1989).

50. F. Yuan, S. Liu, J. Zhou, S. Wang, Y. Wang, S. Xuan, X. Gong, Smart touchless triboelectric nanogenerator towards safeguard and 3D morphological awareness. *Nano Energy* **86**, (2021).

51. Y. Xi, J. Hua, Y. Shi, Noncontact triboelectric nanogenerator for human motion monitoring and energy harvesting. *Nano Energy* **69**, (2020).

52. Y. Tang, H. Zhou, X. Sun, N. Diao, J. Wang, B. Zhang, C. Qin, E. Liang, Y. Mao, Triboelectric Touch‐Free Screen Sensor for Noncontact Gesture Recognizing. *Adv. Funct. Mater.* **30**, (2019).

53. H. Guo, X. Jia, L. Liu, X. Cao, N. Wang, Z. L. Wang, Freestanding Triboelectric Nanogenerator Enables Noncontact Motion-Tracking and Positioning. *ACS Nano* **12**, 3461-3467 (2018).

54. S. Peng, Y. Feng, Y. Liu, M. Feng, Z. Wu, J. Cheng, Z. Zhang, Y. Liu, R. Shen, D. Wang, New blind navigation sensor based on triboelectrification and electrostatic induction. *Nano Energy* **104**, (2022).

55. W. Zhang, Y. Lu, T. Liu, J. Zhao, Y. Liu, Q. Fu, J. Mo, C. Cai, S. Nie, Spheres Multiple Physical Network-Based Triboelectric Materials for Self-Powered Contactless Sensing. *Small* **18**, e2200577 (2022).

56. S. M. S. Rana, M. A. Zahed, M. T. Rahman, M. Salauddin, S. H. Lee, C. Park, P. Maharjan, T. Bhatta, K. Shrestha, J. Y. Park, Cobalt‐Nanoporous Carbon Functionalized Nanocomposite‐Based Triboelectric Nanogenerator for Contactless and Sustainable Self‐Powered Sensor Systems. *Adv. Funct. Mater.* **31**, (2021).

57. D. V. Anaya, K. Zhan, L. Tao, C. Lee, M. R. Yuce, T. Alan, Contactless tracking of humans using non-contact triboelectric sensing technology: Enabling new assistive applications for the elderly and the visually impaired. *Nano Energy* **90**, (2021).

58. F. Wang, Z. Ren, J. Nie, J. Tian, Y. Ding, X. Chen, Self‐Powered Sensor Based on Bionic Antennae Arrays and Triboelectric Nanogenerator for Identifying Noncontact Motions. *Advanced Materials Technologies* **5**, (2019).

59. C. Zhao, Z. Wang, Y. Wang, Z. Qian, Z. Tan, Q. Chen, X. Pan, M. Xu, Y. C. Lai, MXene‐Composite‐Enabled Ultra‐long‐Distance Detection and Highly Sensitive Self‐Powered Noncontact Triboelectric Sensors and Their Applications in Intelligent Vehicle Perception. *Adv. Funct. Mater.* **33**, (2023).

60. J. Cao, X. Fu, H. Zhu, Z. Qu, Y. Qi, Z. Zhang, Z. Zhang, G. Cheng, C. Zhang, J. Ding, Self-Powered Non-Contact Motion Vector Sensor for Multifunctional Human-Machine Interface. *Small Methods* **6**, e2200588 (2022).

61. L. Gao, J. Li, Z. Wang, M. Bu, L. Zhai, S. Wu, N. Hu, K. Dai, L. Wu, A. Lee, X. Mu, A high performance triboelectric nanogenerator based on ordered doping technique for human-machine interaction sensing. *Nano Energy* **95**, (2022).
